# Effect of Extracts, Fractions, and Isolated Molecules of *Casearia sylvestris* to Control *Streptococcus mutans* Cariogenic Biofilm

**DOI:** 10.3390/antibiotics12020329

**Published:** 2023-02-04

**Authors:** Sabrina M. Ribeiro, Paula C. P. Bueno, Alberto José Cavalheiro, Marlise I. Klein

**Affiliations:** 1 Department of Dental Materials and Prosthodontics, School of Dentistry, São Paulo State University (UNESP), Araraquara 14801-903, SP, Brazil; 2 Department of Organic Chemistry, Institute of Chemistry, São Paulo State University (UNESP), Araraquara 14800-900, SP, Brazil; 3 Department of Physics and Chemistry, Faculty of Pharmaceutical Sciences of Ribeirão Preto, University of São Paulo (USP), Ribeirão Preto 14040-903, SP, Brazil; 4 Department of Organic Chemistry, Institute of Chemistry, Federal University of Alfenas, Alfenas 37130-001, MG, Brazil; 5 Department of Oral Diagnosis, Piracicaba Dental School, State University of Campinas (UNICAMP), Piracicaba 13414-903, SP, Brazil

**Keywords:** *Streptococcus mutans*, biofilm, extracellular matrix, dental caries, *Casearia sylvestris*

## Abstract

The effects of extracts, fractions, and molecules of *Casearia sylvestris* to control the cariogenic biofilm of *Streptococcus mutans* were evaluated. First, the antimicrobial and antibiofilm (initial and pre-formed biofilms) in prolonged exposure (24 h) models were investigated. Second, formulations (with and without fluoride) were assessed for topical effects (brief exposure) on biofilms. Third, selected treatments were evaluated via bacterium growth inhibition curves associated with gene expression and scanning electron microscopy. In initial biofilms, the ethyl acetate (AcOEt) and ethanolic (EtOH) fractions from Brasília (BRA/DF; 250 µg/mL) and Presidente Venceslau/SP (Water/EtOH 60:40 and Water/EtOH 40:60; 500 µg/mL) reduced ≥6-logs vs. vehicle. Only the molecule Caseargrewiin F (CsF; 125 µg/mL) reduced the viable cell count of pre-formed biofilms (5 logs vs. vehicle). For topical effects, no formulation affected biofilm components. For the growth inhibition assay, CsF yielded a constant recovery of surviving cells (≅3.5 logs) until 24 h (i.e., bacteriostatic), and AcOEt_BRA/DF caused progressive cell death, without cells at 24 h (i.e., bactericidal). CsF and AcOEt_BRA/DF damaged *S. mutans* cells and influenced the expression of virulence genes. Thus, an effect against biofilms occurred after prolonged exposure due to the bacteriostatic and/or bactericidal capacity of a fraction and a molecule from *C. sylvestris*.

## 1. Introduction

Dental caries is a chronic and multifactorial condition that results from the formation of a polymicrobial biofilm and dynamic interactions between microorganisms present in this biofilm, salivary constituents, and dietary carbohydrates (e.g., sucrose) [1,2]. Despite attempts to raise awareness, this oral condition is a worrying public health problem and impairs the quality of life of millions of people [1]. *Streptococcus mutans* is the bacterium associated with dental caries etiology (although other microorganisms may be associated) [2,3]. *S. mutans* is acidogenic, aciduric, and the leading producer of extracellular matrix in biofilms, known as dental plaque [4]. This trait occurs because *S. mutans* encodes multiple exoenzymes (e.g., glycosyltransferases or Gtfs), and in the presence of sucrose (and starch), they produce copious amounts of exopolysaccharides (i.e., mainly glucans but also fructans) [4,5].

The extracellular matrix provides a cohesive and acidic environment of limited diffusion [6], restricting access to buffering saliva and antimicrobial agents [6,7]. In cariogenic biofilms, exopolysaccharides are primordial components in the organization of the extracellular matrix [4,5] and are determinants of virulence [6]. In addition to exopolysaccharides, extracellular DNA (eDNA) and lipoteichoic acids (LTA) are found in large amounts in cariogenic biofilms [8] and contribute to the matrix structural organization and properties [9,10]. These virulence factors modulate the pathogenesis of dental caries and, thus, are selective therapeutic targets for preventing this disease.

Fluoride, in its various modalities of administration, is the basis for caries prevention; however, its current delivery forms are insufficient to overcome the cariogenic challenges in many individuals; therefore, additional approaches are needed to increase its effectiveness [11]. Chemical agents (such as chlorhexidine?CHX) are widely used to control cariogenic biofilms [12]. Although CHX can suppress mutans group streptococci levels, its efficacy is reduced against mature biofilms, mainly because the exopolysaccharides of the matrix have a negative charge and affect the penetration of CHX (a cationic substance) into the biofilm, compromising its antimicrobial activity in these biofilms [6,12]. In addition, CHX eliminates oral bacteria that convert nitrate to nitrite, which can raise systolic blood pressure [13]. Therefore, CHX cannot be used for a continuous and prolonged period [13]. Natural products are a vast source of structurally diverse molecules with various biological properties. Therefore, natural antibacterial substances are useful for developing alternative or adjunctive anticaries therapies. For example, plant extracts have recently been incorporated into these products to improve their antimicrobial properties [14]. 

*Casearia sylvestris* Sw. (Salicaceae) is one of the most promising species from the genus *Casearia* due to its biological properties and uses in folk medicine. *C. sylvestris* has a high adaptive capacity and is widely disseminated in Central and South America, and in Brazil, it occurs in practically all biomes [15]. Chemically, extracts from leaves of *C. sylvestris* var. *sylvestris* (Atlantic Forest) are rich in diterpenes (taxonomic markers for the genus) [16], while phenolic compounds (flavonoids) predominate in var. *lingua* (Cerrado) [17]. The range of variations around a basic skeleton found in the diterpenes and flavonoids of *C. sylvestris* provides very interesting models for studies on the relationship between chemical structure and biological activity of these compounds, which have already demonstrated potential activity in the control of cariogenic biofilms [18].

Studies about different *C. sylvestris* varieties and their chemical constituents have pointed out a plethora of biological activities such as cytotoxic, anti-inflammatory, and anti-tumor effects, among others [18,19,20,21]. However, few studies have been carried out at a deep level regarding its antimicrobial potential [22,23], and little is clarified about its biological activity against pathogenic microorganisms found in the oral cavity [24]. Thus, prospective studies of plant extracts and/or isolated molecules with antimicrobial and antibiofilm properties are relevant for dentistry and other areas. Therefore, this in vitro study evaluated the effect of extracts, fractions, and isolated molecules of *C. sylvestris* (Atlantic and Cerrado Biomes; *sylvestris*, *lingua*, and intermediate varieties) to control the cariogenic biofilm of *S. mutans*.

## 2. Results

### 2.1. Antimicrobial Activity of Crude Extracts

**Figure 1:**
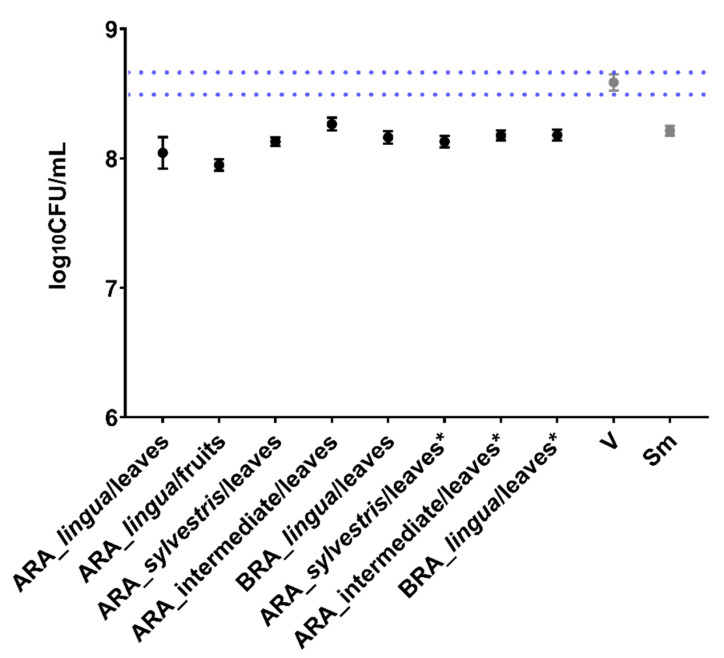
Antimicrobial activity of *C. sylvestris* crude extracts against *S. mutans*. Log_10_ CFU data from planktonic cells treated by crude extracts (500 µg/mL). The central data are the mean, and the error bars are the confidence interval (95% CI). The dotted lines use vehicle data to compare the effectiveness of tested treatments. The growth control (no treatment) is represented as Sm for *S. mutans* and V for the vehicle control (with the concentration in each well being 1.75% EtOH and 0.31% DMSO). The asterisk indicates the raw extracts with the pH adjusted to a value close to the vehicle. The experiments were performed in triplicate on two separate occasions (*n* = 2).

The measure was considered effective when the log_10_ CFU/mL count was reduced by 3 logs (vs. vehicle) [25,26]. Extracts caused mean reductions of 0.5 logs vs. vehicle control (Figure 1); therefore, none inhibited > 3 logs of viable cell counts. Thus, the observed effect is not biologically significant [27]. These data differ from those obtained previously when adequate reduction occurred [18]. These findings demonstrate the requirement to test all batches of extracts to ensure that minimal activity standards are achieved.

### 2.2. Antibiofilm Activity Using the Polystyrene Microplate Model (Prolonged Exposure)

**Figure 2:**
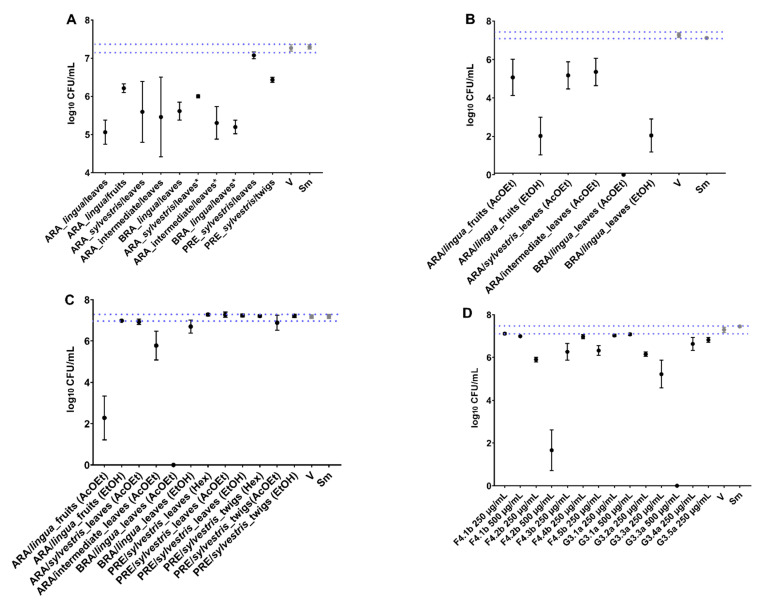
The activity of crude extracts, fractions, and molecules of *C. sylvestris* against initial biofilms. The graphs show in (**A**) log_10_ CFU of the early (24 h) biofilms treated by the crude extracts (500 µg/mL); (**B**) log_10_ CFU of the early (24 h) biofilms treated by fractions obtained via methodology 1 (dry fractions in sample concentrator, 250 µg/mL); (**C**) log_10_ CFU of biofilms treated by fractions obtained via methodology 2 (dry fractions in the fume hood, 250 µg/mL); (**D**) log_10_ CFU of the early (24 h) biofilms treated by SPE-C18 fractions of PRE/SP (250 µg/mL and 500 µg/mL). The central data are the mean, and the error bars are the confidence interval (95%CI). The dotted lines use vehicle data to compare the effectiveness of tested treatments. The growth control (no treatment) is represented as Sm for *S. mutans* and V for the vehicle control (with the concentration in each well being 1.75% EtOH and 0.31% DMSO for the crude extracts, while for the AcOEt, EtOH, and Hex fractions, it was 5.26% EtOH and 0.94% DMSO, finally for the SPE-C18 fractions, 2.63% EtOH). In (**A**), the asterisk indicates the raw extracts with the pH adjusted to the value close to that of the vehicle. The experiments were performed in triplicate on two separate occasions (*n* = 2).

**Figure A1:**
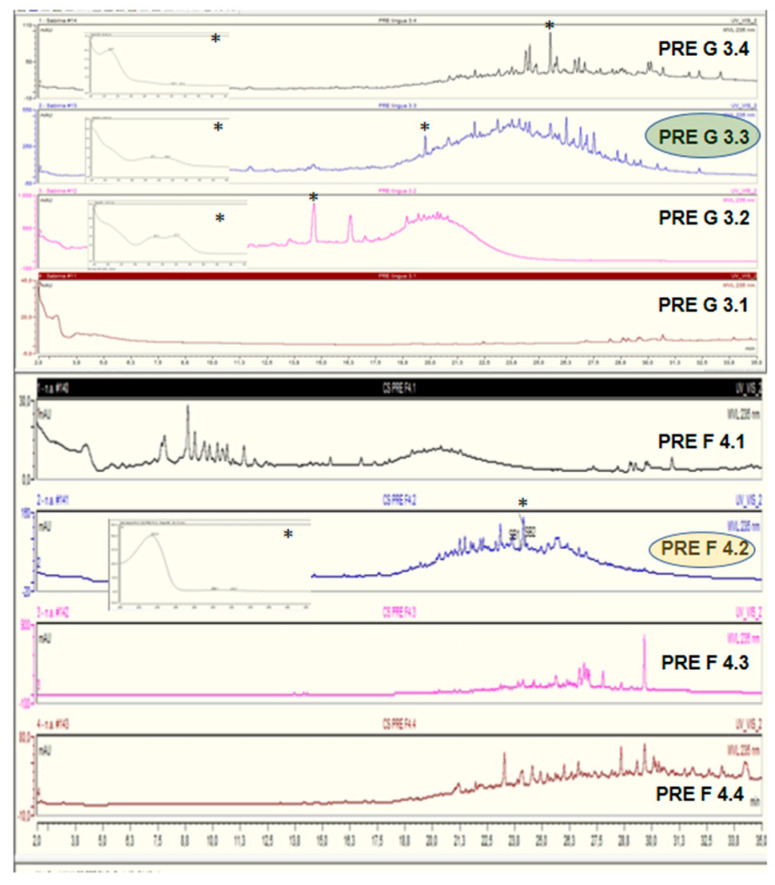
Chemical profile of the SPE-C18 fractions obtained from *C. sylvestris*. The chromatographic profiles of the SPE-C18 fractions indicate that they are rich in clerodane diterpenes and possibly tannins. Asterisks (*) indicate which chromatographic band corresponds to the UV spectrum.

The CFU/mL data obtained in the antibiofilm assays were converted to log_10_ to verify the log reduction. As for the antimicrobial assay, the effect was considered adequate when the log_10_ CFU/mL count was reduced by 3 logs (vs. vehicle) for 24 h old and 48 h old biofilms [25,26]. In early (24 h) biofilms, no extract inhibited > 3 logs of viable cell counts (Figure 2A). Therefore, these extracts did not present good biological activity. All fractions obtained through methodology 1 reduced the viable population count of *S. mutans*. However, considerable reductions occurred for the AcOEt and EtOH fractions of BRA/DF, which decreased 8 and 6 logs of viable cell counts, respectively (vs. vehicle; Figure 2B). For the fractions of methodology 2, AcOEt_BRA/DF reduced 8 logs of viable cell count (vs. vehicle; Figure 2C). None of the Hex fractions caused a considerable reduction (Tables S1 and S2 in the Supplementary Materials). Another attempt to characterize the active fraction was made through the fractionation of extracts from leaves (F) and branches (G) of *C. sylvestris* collected in Presidente Venceslau (PRE) by SPE-C18. The fractions Water/EtOH 60:40 (F4.2b) and Water/EtOH 40:60 (G3.3a), both at 500 µg/mL, reduced, respectively, 6 and 8 logs of the viable cell count of the biofilms (vs. vehicle; Figure 2D). Their chromatographic profiles indicate that they are fractions rich in clerodane diterpenes and possibly tannins (data shown in Figure A1).

**Figure 3:**
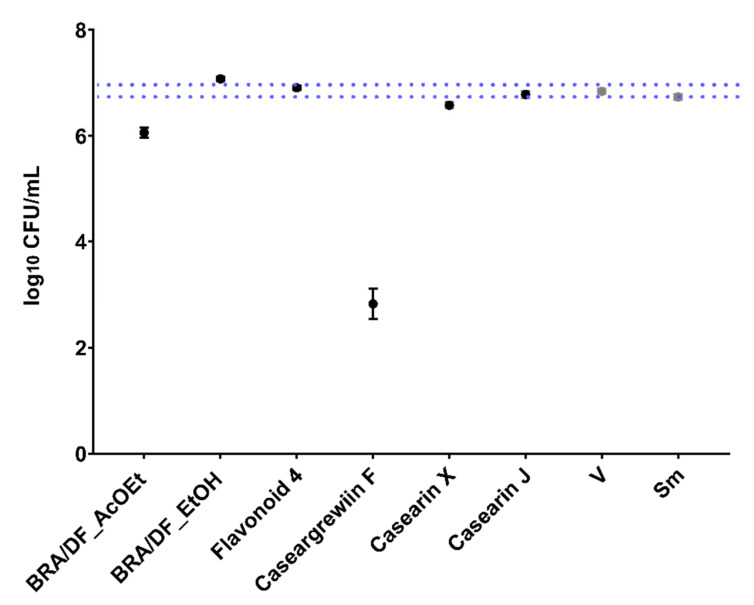
Log_10_ CFU of pre-formed biofilms (48 h old biofilms) treated by fractions (500 µg/mL), flavonoid (125 µg/mL), and casearins (125 µg/mL). The central data are the mean, and the error bars are the confidence interval (95% CI). The dotted lines use vehicle data to compare the effectiveness of tested treatments. The growth control (no treatment) is represented as Sm for *S. mutans* and V for the vehicle control (with the concentration in each well being 1.75% EtOH and 0.31% DMSO). The experiments were performed in triplicate on two separate occasions (*n* = 2).

**Figure A2:**
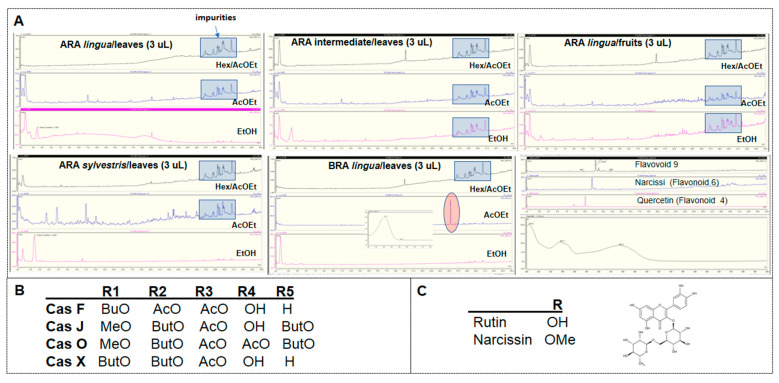
(**A**) The chemical profile of AcOEt fractions from *C. sylvestris* samples. (**B**) Substitutes of casearins, where: BuO = n-C_3_H_7_CO_2_; MeO = OCH_3_ (methoxy); AcO = CH_3_CO_2_ (acetate); OH = O-H (hydroxyl); H = hydrogen. (**C**) Structure of *C. sylvestris* flavonoids and their substituents. The chemical profile of AcOEt was obtained from *C. sylvestris*. The chemical profile of the AcOEt fractions for most samples of *C. sylvestris* showed bands that could not be related to the main secondary metabolites already described for this species, that is, flavonoids and clerodane diterpenes, with the exception of the AcOEt fraction obtained from the AcOEt extract leaves of *C. sylvestris* collected in Brasilia/DF. This fraction is enriched in a constituent whose UV spectrum is characteristic of casearins, with λmax at 241 nm.

Among the four isolated molecules tested, only CsF (125 µg/mL) reduced the viable cell count of pre-formed biofilms vs. vehicle (reduced ≅ 5 logs; Figure 3). The BRA/DF fractions (500 µg/mL) did not interfere adequately with the viability of *S. mutans* in the treated biofilms. For the samples of *C. sylvestris* obtained by SPE-Si/C, the chemical profile showed bands that could not be related to the main secondary metabolites already described for this species, that is, flavonoids and clerodane diterpenes, except for the AcOEt fraction obtained from the extract of leaves of *C. sylvestris* collected in Brasilia-DF (BRA/DF). This fraction is enriched in a constituent whose UV spectrum is characteristic of casearins, with λmax at 241 nm. The relationship of clerodane diterpenes with antibiofilm activity was confirmed by the data presented in Figure 3 for CsF, where the activities of the AcOEt_BRA/DF fraction and patterns of glycosylated flavonoids and casearins are confronted. For the AcOEt _BRA/DF fraction, chromatography identified the main peak, indicating a casearin. However, this peak differs at this time from the peaks of the other casearins used in this study (F, X, and J; chromatographic data shown in Figure A2). Therefore, we would hypothesize that in AcOEt_BRA/DF, there is the presence of an unidentified casearin. 

### 2.3. Effect of Topical Treatments on Biofilms Grown on Saliva-Coated Hydroxyapatite (sHA) Discs

#### 2.3.1. pH of the Spent Culture Medium

**Figure 4:**
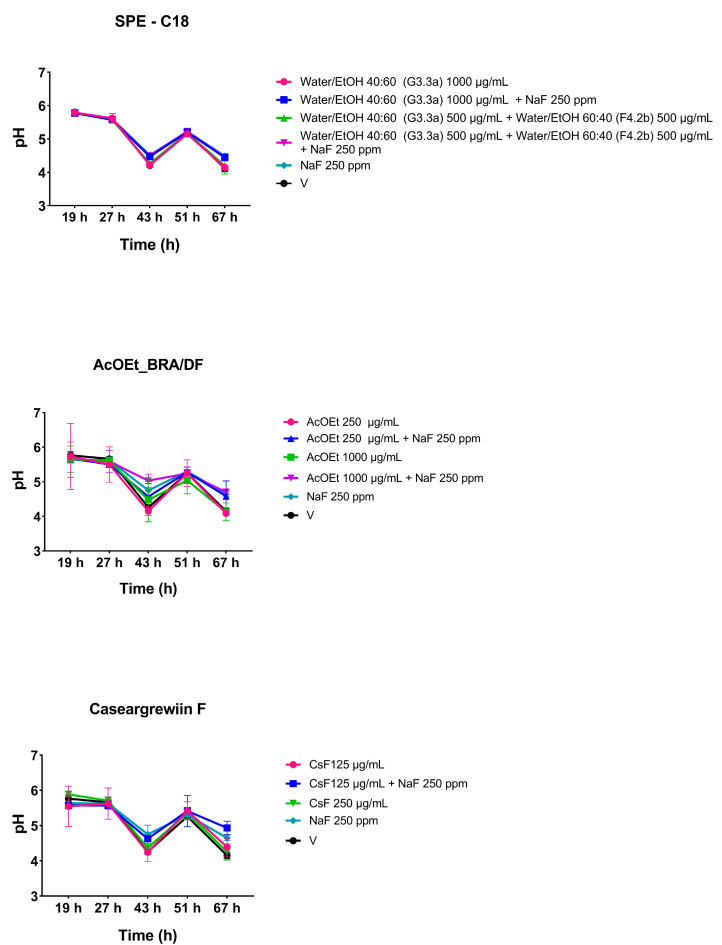
pH of spent culture medium from topically treated biofilms at different developmental stages. The spent biofilm culture media were analyzed at 19, 27, 43, 51, and 67 h. The central data are the mean, and the error bars are the confidence interval (95% CI). The vehicle control is represented as V (with the concentration being 10.52 % EtOH for the SPE-C18 fractions, 21.04% EtOH and 3.75% DMSO for the AcOEt_BRA/DF fractions, and 10.52% EtOH and 1.87% DMSO for CsF). The experiments for SPE-C18, AcOEt_BRA/DF, and CsF 125 µg/mL were performed in duplicate on one experimental occasion (*n* = 1) and in duplicate on two separate occasions (*n* = 2) for CsF 250 µg/mL.

The pH data are represented in Figure 4. These values reflect acidogenicity, measuring the concentration of free hydrogen ions in the medium. However, they do not show whether acid concentration occurred at any specific location within the biofilm or at the interface between the biofilm and the substrate (HA disc). The mean values for 43, 51, and 67 h old biofilms are lower or more acidic compared to 19 h old biofilms. All values are below 5.5, a pH considered critical for demineralizing dental enamel. However, in general, there is a similarity in the pH values between the evaluated groups over time, although the fluoride formulations present higher values.

#### 2.3.2. Bacterial Population and Biofilm Dry Weight (Insoluble Dry Weight)

**Figure 5:**
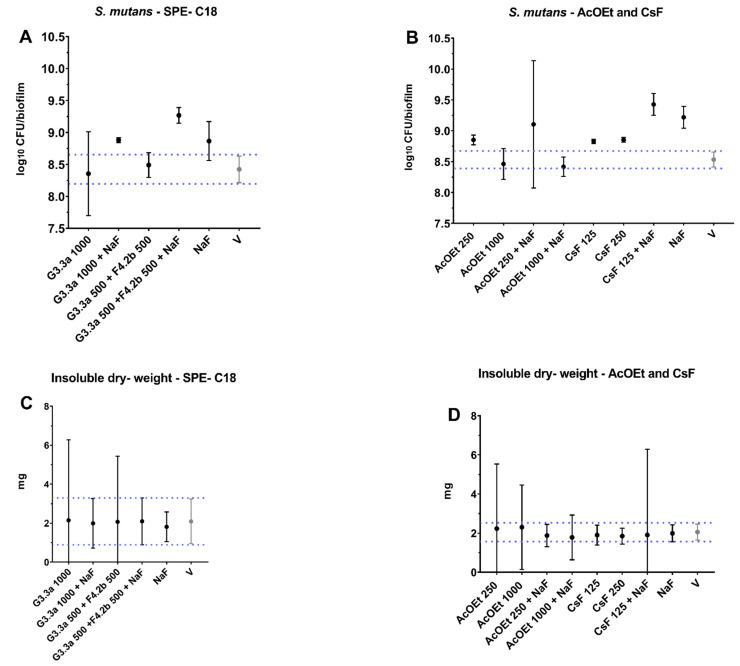
Characterization of biofilms after topical treatments. (**A**) Bacterial population (log_10_ CFU/biofilm) of the SPE-C18 fractions; (**B**) bacterial population (log_10_ CFU/biofilm) of BRA/DF_AcOEt and Caseargrewiin F (CsF) fractions; (**C**) dry weight (mg) for biofilms treated with SPE-C18 fractions; (**D**) dry weight (mg) for biofilms treated with BRA/DF_AcOEt and Caseargrewiin F. The central data are the mean, and the error bars are the confidence interval (95% CI). The dotted lines use vehicle data to compare the effectiveness of tested treatments. The vehicle control is represented as V (with the concentration being 10.52% EtOH for the SPE-C18 fractions, 21.04% EtOH and 3.75% DMSO for the AcOEt_BRA/DF fractions, and 10.52% EtOH and 1.87% DMSO for CsF). The experiments for SPE-C18, AcOEt_BRA/DF, and CsF 125 µg/mL were performed in duplicate on one experimental occasion (*n* = 1) and in duplicate on two separate occasions (*n* = 2) for CsF 250 µg/mL.

All groups were compared to the vehicle control. Topical exposure (1.5 min for SPE-C18 fractions and 10 min for AcOEt_BRA/DF and CsF) affected only the bacterial population, with an increase for Water/EtOH 40:60 (G3.3a) 1000 µg/mL + NaF250 ppm, Water/EtOH 40:60 (G3.3a) 500 µg/mL + Water/EtOH 60:40 (F4.2b) 500 µg/mL + NaF250 ppm (Figure 5A) and at the lowest concentrations tested for the AcOEt_BRA/DF 250 fraction µg/mL and 125 µg/mL CsF combined with NaF (Figure 5B); this also occurred for the control NaF vs. vehicle (Figure 5A,B). There was no pronounced effect on insoluble dry weight (Figure 5C,D).

#### 2.3.3. Components of the Extracellular Matrix of Biofilms

**Figure 6:**
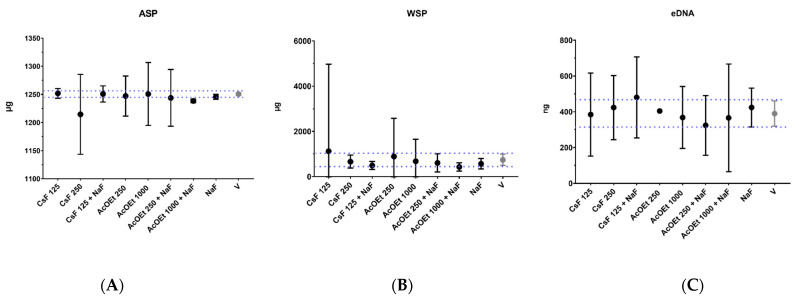
Components of the extracellular matrix of *S. mutans* biofilms after topical treatments. The graphs show the quantification of (**A**) ASP (μg), (**B**) WSP (μg), and (**C**) eDNA (ƞg) of biofilms (formed on sHA discs) after treatment with selected agents and controls. The central data are the mean, and the error bars are the confidence interval (95% CI). The dotted lines use vehicle data to compare the effectiveness of tested treatments. The vehicle control is represented as V (with the concentration being 21.04% EtOH and 3.75% DMSO for the AcOEt_BRA/DF fractions and 10.52% EtOH and 1.87% DMSO for CsF). The experiments for SPE-C18, AcOEt_BRA/DF, and CsF 125 µg/mL formulations were performed in duplicate on one experimental occasion (*n* = 1) and duplicate on two separate occasions (*n* = 2) for CsF 250 µg/mL.

The amount of insoluble (ASP, recovered in the insoluble portion of the biofilm matrix) and soluble (WSP) exopolysaccharides and eDNA (recovered in the soluble portion of the biofilm matrix) are represented in Figure 6. There was no pronounced effect for these extracellular matrix components. Therefore, the data indicate that the antibiofilm effect found in the plate-bottom model may have been mostly due to prolonged exposure (24 h of exposure during initial biofilm formation or 24 h after initial biofilm formation), and this effect may be due to an antibacterial action (cell death; see data in Section 2.4).

#### 2.3.4. Three-Dimensional Structure of Biofilms Using Confocal Microscopy

**Figure 7:**
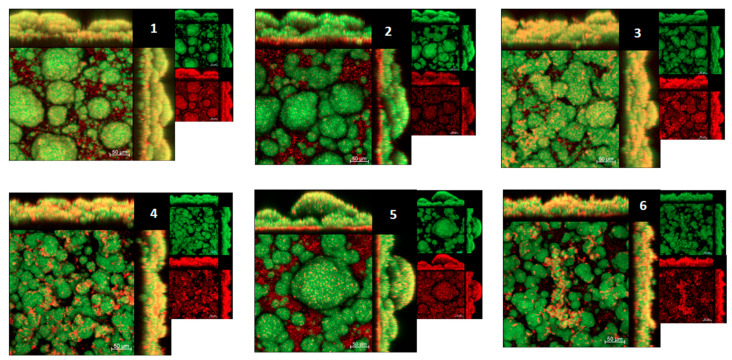
3D architecture of *S. mutans* biofilm grown on sHA discs topically treated with SPE-C18 fractions. Representative confocal images of 67 h old biofilms are displayed in this figure. The red color represents exopolysaccharides produced by *S. mutans* (Alexa Fluor 647), and the green color represents bacterial cells (SYTO 9). The larger image in each set represents the overlay images of the smaller red and green channels (50 μm scale bars), which are shown separately at a smaller size. 1. Water/EtOH 40:60 (G3.3a)—1000 µg/mL; 2. Water/EtOH 40:60 (G3.3a)—1000 µg/mL + NaF 250 ppm; 3. Water/EtOH 60:40 (F4.2b)—500 µg/mL + Water/EtOH 40:60 (G3.3a)—500 µg/mL; 4. Water/EtOH 60:40 (F4.2b)—500 µg/mL + Water/EtOH 40:60 (G3.3a)—500 µg/mL + NaF 250 ppm; 5. NaF 250 ppm; 6. Vehicle (10.52% EtOH).

Figure 7 shows representations of the 3D structure of biofilms on the surface of sHA discs. In the images of biofilms treated with the vehicle control, there are large and defined clusters of microcolonies (in green) protected by exopolysaccharides in the extracellular matrix (in red). The experimental treatments (alone or in combination with NaF) showed similar structural conformation. However, in addition to large microcolonies, there are small microcolonies (among the largest) scattered on the surface of the disc. Finally, for the fluorine-treated (NaF) biofilms, clusters of microcolonies similar to the vehicle-treated biofilms were observed. Therefore, the treatments did not interfere with the three-dimensional organization associated with biofilm virulence.

### 2.4. Growth Inhibition Curve of CsF and AcOEt_BRA/DF and Three-Dimensional Structure of Planktonic Cultures Using SEM

**Figure 8:**
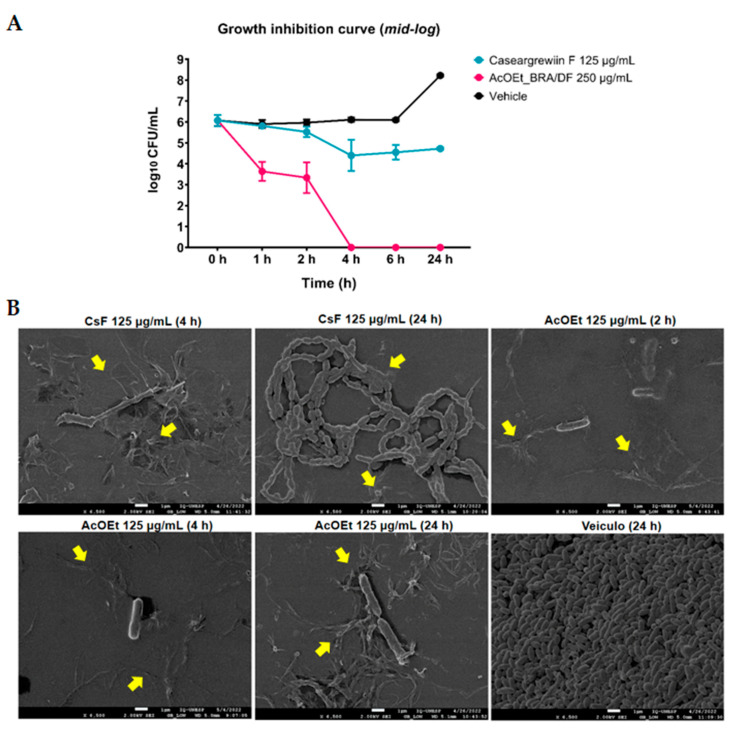
Effects of AcOEt and CsF on planktonic cultures of *S. mutans*. (**A**) The central data for each time, for each evaluated treatment, are the means and the confidence interval (CI 95%) for error bars; (**B**) representative SEM images of *S. mutans* planktonic cells after different exposure times to CsF and AcOEt. The arrows indicate amorphous material from ruptured cells. Scale bars are 1 µm. The concentration of the control vehicle in each well was 5.26% EtOH and 0.94% DMSO.

The data obtained for the growth inhibition curve in planktonic culture are represented in Figure 8A. The effect of AcOEt 250 µg/mL is more pronounced (effective in killing *S. mutans*), with substantial bacterial death after 1 h of exposure (2 logs vs. vehicle), with complete elimination of bacteria within 4 to 24 h of exposure. This effect of AcOEt 250 µg/mL may be due to its composition (presence of an unidentified casearin; Figure A2) or even the concentration used. Importantly, AcOEt 250 µg/mL resulted in 1 log reduction in bacterial population vs. the vehicle in a 48 h biofilm (pre-formed biofilm) and complete elimination of the bacteria in an initial 24 h biofilm. In contrast, there was gradual death from 1 h for CsF 125 µg/mL, and in 4 h, the mean reduction compared to the vehicle control was 1.71 logs. The average recovery of surviving colonies (cells) for CsF was constant from 4 to 24 h, the average difference in 24 h being 3.5 logs compared to the control (vehicle). These data show that the effect of CsF was bacteriostatic, while that of AcOEt was bacteriocidal. Thus, CsF and AcOEt need to be in contact with *S. mutans* cells for much longer than the duration of topical applications (experimentally in this study and recommended for formulations for chemical control of oral biofilms). SEM analysis showed that both AcOEt and CsF promoted the disruption of planktonic *S. mutans* cells (Figure 8B). The images show the presence of amorphous material originating from the ruptured cells, with remnants of the cell wall and released intracellular content (arrows). The surface topography of vehicle-control-treated cultures shows that the vehicle used did not affect *S. mutans* cell morphology. 

### 2.5. Gene Expression

**Figure 9:**
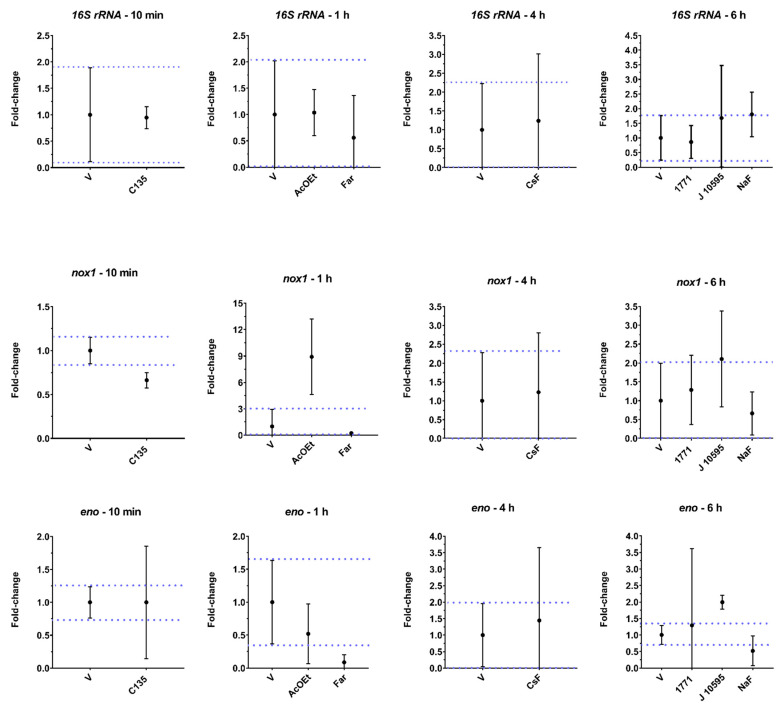

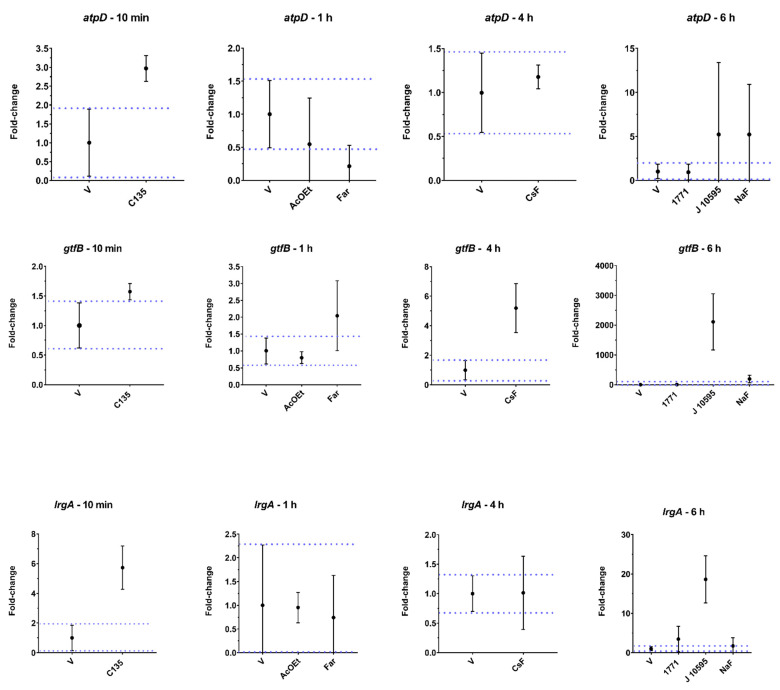
*S. mutans* gene expression of planktonic cultures treated with distinct agent. The central data are the mean, and the error bars are the confidence interval (95% CI). The dotted lines use vehicle data to compare the effectiveness of tested treatments. For detailed information, see the graph of *gtfB* at 6 h for 1771 vs. V in Figure A3.

The effect of seven selected treatments on *S. mutans* gene expression is depicted in Figure 9. Besides choosing the AcOEt fraction and molecule CsF, other compounds with information on possible targets in *S. mutans* cells were tested. Data were obtained on one experimental occasion and quantified by qPCR in triplicate. Genes were not normalized by *16S rRNA* because there was a difference in expression between flavonoid myricetin (J10595) and NaF vs. vehicle. Thus, the normalization was performed by using the same amount of RNA used for cDNA synthesis. *16S rRNA* presented a higher expression after treatment with myricetin (J10595) and NaF. For the *nox1*, the expression was repressed by hydroxychalcone C135 and induced by AcOEt and myricetin (J10595) (vs. vehicle). For *eno*, a substantial reduction in the expression occurred after AcOEt, *tt*-farnesol (Far; a terpenoid), and NaF, while myricetin (J10595) increased the expression of this gene. Exposure to C135, myricetin (J10595), and NaF increased *atpD* expression, while exposure to *tt*-farnesol repressed *atpD* (vs. vehicle). The *gtfB* gene, which encodes an exoenzyme that synthesizes insoluble exopolysaccharides (glucans), showed higher expression for C135, *tt*-farnesol, CsF, myricetin (J10595), and NaF (vs. vehicle). In contrast, it appears that AcOEt reduces the expression of this gene. The gene *lrgA* was induced after treatment with C135, compound 1771 (an inhibitor of LTA metabolism), and myricetin (J10595). This finding demonstrates that the product of this gene, LrgA, which is responsible for coordinating the remodeling of the cytoplasmic membrane (a process that releases DNA to the extracellular milieu), can be affected directly or indirectly because the agents may have interfered with the fluidity of this membrane.

## 3. Discussion

*C. sylvestris* has a phytochemical composition marked by clerodane-types diterpenes and glycosylated flavonoids [17,18,20,28,29]. Prospection studies of this plant have shown activity against cariogenic biofilms. Therefore, it is of interest to the community that its biological properties be explored. Our results indicate that Caseargrewiin F (CsF) and the AcOEt fraction obtained from the leaves of sample BRA/DF (corresponding to variety *lingua* from the Cerrado biome) are effective in inhibiting *S. mutans* through irreversible damage to its structure and changes in the expression of specific virulence genes. However, the topical application does not exert any activity on *S. mutans* biofilm components (viable population, biomass, extracellular matrix components, and structural organization). These findings indicate that the antibiofilm effect in the polystyrene plate-bottom models may have been mainly due to prolonged exposure (24 h) during (the initial biofilm) or after biofilm formation (on pre-formed biofilms). Moreover, the activity of these treatments was primarily due to antibacterial action, verified in the growth inhibition assay. In contrast to previous findings [18], here, no crude extract of *C. sylvestris* showed activity against the long-exposure (24 h) models (Figure 2), which demonstrates that the chemical composition of secondary metabolites is closely related to geographic location, seasonal effects, and biological diversity and drastically influences the biological activity of samples [18,30].

In this long-exposure model, the AcOEt_BRA/DF and SPE-C18 fractions of PRE/SP (Water/EtOH 60:40-F4.2b and Water/EtOH 40:60-G3.3a) reduced the population of the initial biofilms. Additionally, of the isolated molecules evaluated, only CsF showed activity against pre-formed biofilms. CsF is a clerodane-type diterpene with the molecular formula C_28_H_40_O_8_, which was previously isolated from the ethanolic extract of *C. sylvestris* leaf [20]. The chemical structure of CsF seems relevant for its biological activity against *S. mutans*. The presence of OAc at the R2 and R3 terminals of CsF could influence its activity since the exchange for OBu (Casearin X-C_32_H_46_O_9_) and OMe (Casearin J-C_31_H_44_O_9_) in R1 and OBu in R2 (Casearin X and J) seems to significantly inhibit casearin activity. No effect was observed for Casearin X and Casearin J, consistent with the hypothesis of radical terminal placement and molecule configuration for CsF exerting its impact on the bacterium cell. Nonetheless, when tested for topical effects (brief exposure) on *S. mutans* biofilms, these treatments (SPE-C18, AcOEt_BRA/DF, and CsF) did not inhibit biofilm components (Figure 5, Figure 6 and Figure 7). Thus, for such agents to exert their effect, it would be necessary to prepare formulations capable of retaining their active principle in the oral cavity for the time required for their action. Therefore, in the future, these treatments could be used for loading in drug delivery systems (suitable for the oral cavity) to prolong the exposure time at an adequate concentration [31,32].

In addition to the need for a prolonged exposure time for potential activity (see Figure 2, Figure 3 and Figure 8A), some substances/molecules with antimicrobial activity may not be an antibiofilm agent, and a compound with antibiofilm activity (e.g., effect on inhibiting microbial adhesion and/or extracellular matrix build-up) may not be an antimicrobial per se [33]. Here, this behavior was observed for AcOEt_BRA/DF and CsF. Although brief exposure to these treatments had no activity, when evaluated through the *S. mutans* growth inhibition curve, CsF interfered with the viability of *S. mutans* after 1 h of contact, and the mean recovery of surviving cells was constant until the 24 h test, which demonstrates a bacteriostatic effect. However, the bacterial survival profile obtained for AcOEt_BRA/DF showed cell death after 1 h of exposure, with a bactericidal effect (Figure 8A). Therefore, the isolated molecule would be expected to be more effective in eradicating the microorganisms than the fraction. However, isolated molecules are often not as effective as active fractions because the biological activity of the whole natural product results from the synergistic or additive interactions of different compounds in the mixture and not of a single active molecule [30]. SEM analysis demonstrates that these treatments disrupt *S. mutans* cells (in planktonic culture; Figure 8B), which indicates that the antimicrobial mechanism is mainly related to irreversible damage to the microbial cytoplasmic membrane.

Thus, planktonic cells of *S. mutans* were also treated with different agents with recognized targets (compound 1771, C135, myricetin, *tt*-farnesol, and NaF) and, after, evaluated for the expression of virulence genes via the gene expression profile to understand the possible mechanisms of action involved in the biological activity observed for CsF and AcOEt_BRA/DF. Exposure to the flavanoid myricetin (J10595) induced *eno* and *atpD* gene expression. This induction may have occurred because *S. mutans* has developed adaptive acid tolerance responses through the induction of multiple cellular pathways to tolerate the acidification of the acidic environment it produces during glucose consumption [34]. One of these defense pathways against environmental challenges, such as acid shock, is the bacterial cytoplasmatic membrane itself [34]. The *atpD* gene encodes a functional subunit of the F_0_F_1_-ATPase system that is membrane-bound and important for the survival of *S. mutans* under acidic stress. Therefore, induction of *atpD* expression demonstrates that cells reacted to intracellular (cytoplasmic) acidification caused by increased glycolytic activity (increased eno expression), and this induced *atpD* expression [35].

The gene *gtfB* encodes the GtfB enzyme, which metabolizes sucrose into water-insoluble glucans [36]. Here, we observed that CsF, *tt*-farnesol, and myricetin (J10595) induced the expression of this gene. This result was unexpected because the medium contained glucose and the substrate of the GtfB enzyme is sucrose to synthesize glucans, demonstrating that the microorganism was expressing a crucial gene for its survival in a cariogenic biofilm. It may be that the agents promote stress to bacterial cells in their free form (planktonic), and in response to this condition, the microorganism responds with an increase in the gene expression of *gtfB* as an attempt to produce glucans on the cell surface or to adhere to a surface to protect the cell from the stressor. However, myricetin (J10595) is an effective inhibitor of *gtfB* expression in solution, whereas *tt*-farnesol targets the cytoplasmatic membrane, decreasing acid tolerance and the Gtfs enzymes of *S. mutans* [37,38]. Thus, it appears that cells in biofilm and cells in their free form are affected differently by agents. Therefore, the *gtfB* expression profile here (Figure 9) differs from previous ones, where the agents were tested on biofilms through brief exposure [37,38]. Furthermore, inhibition/induction of gene expression and enzyme activity are not always correlated because several post-transcriptional regulatory processes can occur after mRNA is produced (as reviewed before [39].

For *lrgA*, there was an increase in gene expression after treatment with hydroxychalcone C135, compound 1771 (LTA metabolism inhibitor that affects the composition of the Gram-positive cell wall), and myricetin (J10595). The induction of *lrgA* expression and in the expression of genes related to membrane alterations (e.g., induction of *atpD* expression, observed for C135) demonstrate that these agents compromise the ability of *S. mutans* since LrgA (product of *lrgA*) is a membrane-associated protein and controls autolysis and cell death by modulating bacterial cell wall permeability [40,41]. Furthermore, changes in fatty acid profiles affect F-ATPase function and overall membrane permeability, altering the ability of *S. mutans* to maintain intracellular ΔpH, greatly impairing acid tolerance; this induces *atpD* expression, as observed for C135 and myricetin (J10595).

Changes in *nox1* gene expression alter the fatty acid composition of the microbial membrane and interfere with the activity of its product (Nox) [42]. This finding demonstrates that the Nox product can be directly or indirectly affected by C135, AcOEt_BRA/DF, and myricetin (J10595) through changes in membrane physiology, normal function of enzymes involved in glycolysis (enolase), and exopolysaccharides (GtfB) production because these agents downregulated *eno* expression. Alterations in the expression of this gene have a potentially lethal effect on S. *mutans* [43], as observed by the structural damage caused by AcOEt_BRA/DF (SEM images). Repression of *eno* by *tt*-farnesol (a terpenoid) and NaF was already expected since *tt*-farnesol reduces the glycolytic activity of *S. mutans* [30,37], and it is well established that enolase is a target of fluoride [44]. The *atpD* gene was downregulated after treatment with *tt*-farnesol, and there seems to be a reduction for AcOEt, although the magnitude was not relevant at this exposure time. This finding would reinforce that the treatments cause cytoplasmic acidification, which in turn impairs the normal function of the enzymes involved in glycolysis (enolase), as already demonstrated for *tt*-farnesol [30,37]. This outcome would explain the bactericidal effect of AcOEt and the structural damage, mainly in the cell wall of *S. mutans*, observed by SEM analysis.

CsF is a clerodane-type diterpene, and AcOEt fractions are rich sources of this secondary metabolite (also called casearins) [18]. Therefore, we propose that the findings of this study are closely correlated with this class of secondary metabolites. Studies regarding the chemistry of different diterpenes and the relationship of their molecular geometry versus their effectiveness in inhibiting the growth of *S. mutans* demonstrate that the structural characteristics are fundamental for the antimicrobial activity observed for these metabolites [45]. The antimicrobial properties of diterpenes are associated with their potential to promote bacterial lysis and rupture of the cytoplasmic membrane. This activity occurs through the structural characteristics of diterpenes, which include a lipophilic structure capable of insertion into the cell membrane and a hydrophilic fragment having a hydrogen-bonding donor group, which interacts with phosphorylated groups in the membrane [23]. Therefore, we hypothesize that the biological effect obtained for CsF and AcOEt_BRA/DF is associated with bacteriostatic and/or bactericidal capacity due to alterations in the membrane of *S. mutans* by the terpenoids present in these formulations. Furthermore, agent–membrane interactions may occur during cell division or cell wall remodeling, as these events facilitate the entry and/or interaction of the molecule with the cytoplasmic membrane. Unlike CsF which has bacteriostatic activity, the reason that AcOEt works as a bactericidal agent seems to be related to the presence of a new casearin not yet identified in the literature. Therefore, we are working on additional analyses to identify this metabolite.

The ability to effectively disrupt biofilm-specific and lifestyle-essential pathways of bacterial pathogens, all without affecting the viability of normal flora, is an attractive approach to the prevention and/or reduction of biofilm-related diseases, especially those that occur in complex microenvironments, such as the human mouth [30]. Considering the findings of this study, CsF and AcOEt_BRA/DF bring new and significant perspectives for developing selective antibiofilm/antimicrobial agents for potential applications in the prevention of cavities. Although the details of the cytotoxicity of these agents have not been investigated here, our previous study demonstrated low/moderate toxicity at higher concentrations of crude extracts of *C. sylvestris* in long-term exposure models [18]. In the future, these treatments will be evaluated for their cytotoxic activity through three-dimensional culture models.

Interpretation of in vitro results requires caution to avoid overestimation of observed effects. The rapid screening model on polystyrene plates was used here to select agents with potential antimicrobial and/or antibiofilm activity; however, they do not reflect the complex polymicrobial, ecological, and environmental influences found in the oral cavity. Thus, to verify the properties shown in our conditions, future in vitro tests will be carried out for drug delivery systems to prolong the exposure time at an adequate concentration using a polymicrobial (microcosmos) model. Additionally, recognizing the common ability to interact with the cell membrane of *S. mutans*, we propose as future investigations the simulation of the insertion of these agents in a phospholipid membrane model to validate our conclusions about the structure–activity relationships of this class of compounds [23].

## 4. Materials and Methods

### 4.1. Plant Material

Samples of *C. sylvetris* were collected from four trees from three Brazilian regions belonging to the biomes: Cerrado and Atlantic Forest between September/October 2019 and December 2020 (Table 1; Figure S1). All samples were sent to Instituto Agronômico de Campinas (State of São Paulo, Brazil) for identity confirmation and variety assignment by Profa. Dr. Roseli B. Torres. The plant is registered in the National System for the Management of Genetic Resources and Associated Traditional Knowledge (SisGen; Registration nº A00892A), and the collections were authorized by the Brazilian Institute for the Environment and Renewable Natural Resources (IBAMA) through the Authorization and Biodiversity Information (SISBIO; registration n^o^ 33429-1).

### 4.2. Sample Preparation

The plant materials (fruits, leaves, and/or twigs) were dehydrated (40 °C in a circulating air oven) and then stored protected from light and at room temperature until use. Then, the samples were individually crushed, and 20 g of each sample was used to prepare the crude extracts, as described before [18]. First, the extracts were combined and lyophilized, producing seven lyophilized crude extracts. Then, these extracts were solubilized with 84.15% ethanol (EtOH; Sigma-Aldrich Co. St. Louis, MO, USA) and 15% dimethyl sulfoxide (DMSO; Sigma-Aldrich Co. St. Louis, MO, USA) and stored at −80 °C until biological assays.

The same plant material was used for a different approach to prepare crude extracts (combined and subjected to Speed Vac model SPD-Thermo Scientific to remove the extracting solvent) to optimize fraction yields. Then, two methodologies were adopted for fractionation (SPE-Si/C fractions). In the first method, fractionation was carried out from the lyophilized crude extracts, and then the resulting fractions were dried with a Speed Vac (methodology 1). In the second method, fractionation was performed from the crude extracts dried under a Speed Vac, and then the fractions were dried in a fume hood (methodology 2). The fractionation of crude extracts was conducted as described previously [18]. Briefly, a mixture of 40–63 μm, 60 Å silica gel (Merck, Darmstadt, Germany), and activated carbon (Labsynth, Diadema, Brazil) (1:1) was added to solid phase extraction (SPE) cartridges. Columns were preconditioned with 95:5 ethyl acetate (both from J.T. Baker, HPLC grade), and then 150 mg of samples were applied. First, fractions were eluted with 10 mL of 95:5 (% *v*/*v*) hexane/ethyl acetate (Hex fraction), 100% ethyl acetate (AcOEt fraction), and 100% ethanol (EtOH fraction), respectively. Then, the solvents were evaporated with a Speed Vac or under the fume hood, resulting in their respective dry fractions. Next, the fractions were solubilized with 84.15% EtOH and 15% DMSO and stored at −80 °C until the biological assays.

For the PRE/SP crude extracts, solid phase fractionation was performed using reversed-phase silica (Si-C18) (SPE-C18 fractions). The stationary phase consisted of 30 g of C18 silica, dry packed in a polypropylene tube with an internal diameter of 3.7 cm (Polygoprep^®^ Silica 60–50 C18, 50 µm; Macherey-Nagel). For sample application and elution, the dry crude extract was dispersed in C18 and deposited on the adsorbent. First, the elution was carried out with Water/EtOH in the proportions 95:05, 60:40, 40:60, and 20:80 and then with pure EtOH, with the aid of a vacuum, using about 150 mL of each eluent. Next, the solvents were evaporated with a Speed Vac, and then, fractions were solubilized with 84.15% EtOH and 15% DMSO and stored at −80 °C until biological assays. After the elution of each crude extract and fraction, the working solution of each one was evaluated for their pH value to ensure that all treatments and vehicle control showed no difference concerning this parameter (Table S3 in the Supplementary Materials).

Four compounds were isolated from leaves extracts screened in a previous study [18]: the flavonoid 4 (rutin), and the clerodane diterpenes Caseargrewiin F, Casearins X and J (Table 2). The isolation of molecules was performed as described before [17,28]. Casearin substitutes are represented in Table 3.

Casearin substitutes represented in Table 2 and used in the study, where: OBu = n-C_3_H_7_CO_2_ group; OMe = OCH_3_ (methoxy) group; OAc group = CH_3_CO_2_ (acetate); OH = O-H (hydroxyl) group; H = hydrogen (adapted from [17]).

### 4.3. Bacterium Strain and Growth Conditions

Stocks of *S. mutans* strain UA159 (ATCC 700610) stored at −80 °C (tryptic soy broth containing 25% glycerol; Synth, Diadema, SP, Brazil) were thawed and seeded on blood agar plates (5% blood of sheep; Laborclin, Pinhais, PR, Brazil) and incubated at 37 °C, 5% CO_2_ for 48 h (Thermo Scientific, Waltham, MA, USA). After, starter cultures were prepared using 10 colonies that were inoculated in tryptone-yeast extract broth (TY; 2.5% tryptone, 1.5% yeast extract, pH 7.0; Becton Dickinson and Company, Sparks, MD, USA) containing 1% glucose (Synth, Diadema, SP, Brazil), followed by incubation for 16 h (37 °C, 5% CO_2_). Then, the starter cultures were diluted 1:20 in fresh TY + 1% glucose and incubated until the middle of the logarithmic growth phase (optical density or OD5_40nm_ 0.847 (±0.273) and colony forming units per milliliter (CFU/mL) 1.37 × 10^9^ (±6.10 × 10^7^); Kasvi spectrophotometer, Beijing, China)). Inoculums for the assays described here were prepared with a defined population of 2 × 10^6^ CFU/mL in TY + 1% glucose for antimicrobial assays and TY + 1% sucrose (Synth, Diadema, SP, Brazil) for biofilm assays.

### 4.4. Antimicrobial Activity through Prolonged Exposure Model (24 h) 

Antimicrobial activity was evaluated for crude extracts (500 µg/mL). These concentrations were used due to the yield of extracts and fractions and the scientific literature, which considers adequate studies with 1 mg/mL for extracts or 0.1 mg/mL for isolated molecules [46]. First, 100 µL of *S. mutans* cultures (2 × 10^6^ CFU/mL) were transferred to 96-well microplates (Kasvi, Beijing, China) containing test concentrations of treatments or vehicle (control with diluent of treatments) and culture medium (TY + 1% glucose), totalizing 200 µL (resulting in 1 × 10^6^ CFU/mL). All experiments contained the following controls: wells containing culture medium only, wells containing only the experiment inoculum (microbial growth control), and wells containing the inoculum plus vehicle or 0 µg/mL). Then, the microplates were incubated (24 h, 37 °C, 5% CO_2_). After, a visual analysis of the wells was performed (turbidity: microbial growth; clear: no growth), followed by the reading of OD_562nm_ readings (ELISA plate reader, Biochrom Ez, Cambourne, UK). Furthermore, to determine the viability of the microbial cells, an aliquot from each well was used for a serial 10-fold dilution (10^−1^ to 10^−5^) in microtubes containing saline solution (0.89% NaCl; Química Moderna, Barueri, SP, Brazil) and 10 µL aliquots of each dilution plus undiluted culture were used for plating in duplicate on BHI agar plates (Himedia, Dindhori, Nashik, India) and incubated (48, 37 °C, 5% CO_2_) followed by colony counting. CFU data were transformed into log_10_ and analyzed versus the vehicle control. Each treatment was performed in triplicate on two separate occasions (*n* = 2) [18,29].

### 4.5. Antibiofilm Activity through Prolonged Exposure Models (24 h)

The antibiofilm activity of crude extracts 500 µg/mL, fractions (250 µg/mL and 500 µg/mL), and molecules (125 µg/mL) was investigated [18,46]. This analysis was performed using two treatment exposure settings: (I) activity against initial biofilm formation (incubation of agents with cells from the beginning of biofilm formation until analysis after 24 h) for crude extracts, Hex; AcOEt and EtOH fractions, and SPE-C18 fractions; (II) activity against pre-formed biofilm (24 h old biofilms were exposed to treatments for 24 h, yielding 48 h old biofilms) for AcOEt_BRA/DF and EtOH_BRA/DF, Flavonoid 4, Caseargrewiin F (or CsF), and Casearins X and J.

#### 4.5.1. Activity against Initial Biofilm Formation (24 h Old Biofilms)

For 24 h old biofilms, treatments were introduced at 0 h, and biofilms were evaluated at 24 h of development to assess inhibition of biofilm formation. Biofilms were formed in polystyrene microplate wells to verify the viable population (CFU/biofilm) of bacterium cells in biofilms treated by crude extracts or fractions, as described before [29]. A 96-well plate was prepared as described in item 4.4, including the set of controls. However, here, the culture medium used was TY + 1% sucrose. The plate was incubated (24 h, 37 °C, 5% CO_2_). Then, a visual analysis of wells was performed, and the plate was subjected to orbital shaking (5 min, 75 rpm, 37 °C; Quimis, G816 M20, São Paulo, Brazil). The culture medium with loose cells was aspirated and discarded. The biofilms remaining in the wells were washed (three times) with a pipette and 200 µL of 0.89% NaCl solution to remove non-adhered cells. Next, these biofilms were scraped with pipet tips five times with 200 µL of 0.89% NaCl, totalizing 1 mL of biofilm suspension (from each well). This biofilm suspension was placed in a microtube and subjected to serial dilutions (10^−1^ to 10^−5^), which were plated, as were the undiluted biofilm suspensions. The BHI plates were incubated (48 h, 37 °C, 5% CO_2_), followed by colony counting. Next, data CFU were transformed into log_10_ and analyzed compared to the vehicle control. Two independent experiments were performed in triplicate (*n* = 2).

#### 4.5.2. Activity against Pre-Formed Biofilms (48 h Old Biofilms)

In this setting, the biofilms were formed in polystyrene microplate wells without the addition of any treatment or vehicle control. After 24 h, the formed biofilms were exposed to treatments for 24 h to determine the prevention of biofilm accumulation (48 h biofilms). Then, the biofilms were evaluated at 48 h of development to verify the inhibition of biofilm formation via viable population analysis (CFU/biofilm) [33]. For this evaluation, 50 µL of final inoculum of *S. mutans* (2 × 10^6^ CFU/mL) and 50 µL of TY + 1% sucrose (to obtain 1 × 10^6^ CFU/mL), and 50 µL of TY + 1% sucrose (to reach 1 × 10^6^ CFU/mL) were added to wells of 96-well plates. The microplate was incubated (24 h, 37 °C, 5% CO_2_) without any treatment or vehicle control. After incubation and biofilm formation, visual analysis was performed, followed by culture medium removal and washing of the remaining biofilms (three times with 0.89% NaCl solution). Next, fresh culture medium TY + 1% sucrose and test concentrations of treatments or the vehicle were added. For each experiment, the same controls described in item 4.4 were included. The microplate was incubated again (24 h, 37 °C, 5% CO_2_). After incubation (when biofilms were 48 h old), the same processing protocol applied for 24 h old biofilms was conducted until obtaining 1 mL of biofilm suspension. The biofilm suspensions were sonicated (30 s, 7 w, Sonicator QSonica, Q125, Newtown, CT, USA) and subjected to serial dilutions (10^−1^ to 10^−5^), which were plated in BHI agar (in addition to undiluted biofilm suspensions), followed by incubation (48 h, 37 °C, 5% CO_2_) and colony counting. Next, data CFU were transformed into log_10_ and analyzed compared to the vehicle control. Two independent experiments were performed in triplicate (*n* = 2).

### 4.6. Analysis of the Effect of Topical Application (Brief Exposure Time) of Formulations on Biofilms Formed on sHA Discs

In this step, selected treatments (“promising” data for antibiofilm activity on initial and pre-formed biofilms) were used to prepare formulations (combined with and without sodium fluoride-NaF). These formulations were then tested on sHA discs and biofilms grown on them [47]. The selected fractions were AcOEt_BRA/DF, SPE-C18 Water/EtOH 40:60 (G3.3a), and Water/EtOH 60:40 (F4.2b), and the molecule was CsF.

#### Biofilm Formation on sHA Discs and Topical Treatments

Biofilms of *S. mutans* UA159 were grown on sHA discs (surface area of 2.93 ± 0.2 cm^2^, Clarkson Chromatography Products Inc., South Williamsport, PA, USA) in batch cultures for 67 h, as detailed elsewhere [9]. Saliva and pellicle preparation were performed as described before [48]. Saliva was donated by two volunteers who did not undergo antibiotic therapy in the last three months. The volunteers rinsed their mouths with 5 mL of Milli-Q water, then masticated a portion of parafilm, collecting 5 mL of saliva into a collection tube, which was then discarded. Next, the volunteers continued masticating the parafilm (Parafilm M; Sigma-Aldrich Co., St. Louis, MO, USA) and collected saliva into an ice-chilled Falcon tube. The saliva samples from all volunteers were combined and diluted 1:1 with adsorption buffer (AB buffer: 50 mM KCl, 1 mM KPO_4_, 1 mM CaCl_2_, 1 mM MgCl_2_, 0.1 mM PMSF, in dd-H_2_O, pH 6.5). The samples were centrifuged (3220× *g*, 20 min, 4 °C; Centrifuge 5810R, Eppendorf), and the clarified portion was filtered-sterilized (0.22 µm low protein binding polyethersulfone membrane filter, Rapid-flow Nalgene Thermo Scientific, Monterrey, Mexico). Fresh saliva was used for pellicle formation and culture medium preparation at the start of the experiment. Aliquots of the prepared saliva samples were stored at −80 °C until use for culture media preparation. Then, sHA discs were vertically positioned in wells of a 24-well microtiter plate (Kasvi, Beijing, China) with the help of custom-made disc holders [6]. Next, the sHA discs were dip-washed into wells containing AB buffer and topically treated with the tested treatments or vehicle control, as summarized in Table 4.

The surfaces of each sHA disc were subjected to topical treatment for 1.5 min or 10 min by dripping 300 µL of each treatment or control (two discs per treatment and experiment). Then, the discs were washed by immersion in wells containing AB buffer (to remove excess treatment) and transferred to wells containing *S. mutans* culture for biofilm formation (0 h biofilm formation). The biofilm inoculum was prepared with the cultures of *S. mutans*. After reaching the late exponential growth phase, the cultures were diluted in TY + 0.1% sucrose and 20% saliva (to obtain 2 × 10^6^ CFU/mL). The biofilms were incubated for 6 h (37 °C, 5% CO_2_), when the discs with initial biofilm were rinsed with 0.89% NaCl, treated with each corresponding treatment (as above), rinsed with 0.89% NaCl (to remove excess treatment), and transferred back to the corresponding culture media until biofilms were 19 h old, when culture medium was changed.

The culture medium was changed twice a day until the end of each experimental occasion: at 8 a.m. (TY + 0.1% sucrose and 20% saliva) and 4 p.m. (0.5% sucrose + 1% starch (Sigma-Aldrich Co., St. Louis, MO, USA) and 20% saliva). After each media change, the pH of the spent media was measured. The biofilms were topically treated two hours after each culture change (Figure S2). Biofilms were grown until 67 h for evaluation of bacterial population, biomass, biochemical characteristics of the extracellular matrix, and structure (confocal microscopy).

### 4.7. Biofilm Analyses

#### 4.7.1. Biofilm Processing and Standard Methods of Biochemical Analysis (Colorimetric) and Microbial Culture Method

**Figure A4:**
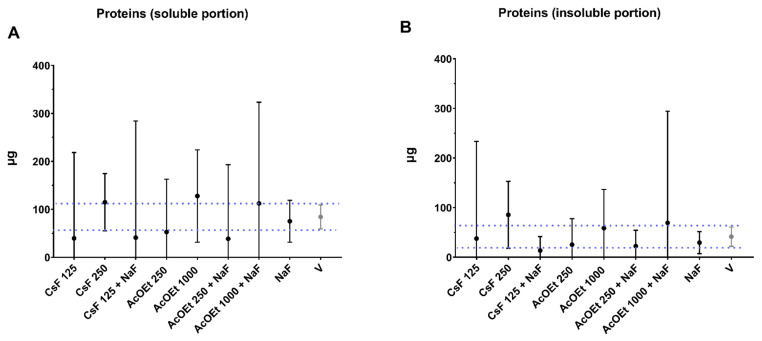
Total protein content (soluble and insoluble portions) of topically treated biofilms. The graphs show the total protein content in the soluble (**A**) and the insoluble portions (**B**) of topically treated biofilms. The central data are the mean, and the error bars are the confidence interval (95% CI). The blue dotted lines use vehicle data to compare the effectiveness of tested treatments. The vehicle control is represented as V (with the concentration being 21.04% EtOH and 3.75% DMSO for the AcOEt_BRA/DF fractions and 10.52% EtOH and 1.87% DMSO for CsF). The experiments for AcOEt_BRA/DF and CsF 125 µg/mL were performed in duplicate on one experimental occasion (*n* = 2) and in duplicate on two separate occasions (*n* = 4) for CsF 250 µg/mL.

When they reached 67 h, the biofilms were processed for analyses following previously described protocols [48]. Standard methods of biochemical analysis (colorimetric) were used to determine total protein content (in the soluble portion and the insoluble portion; Figure A4), exopolysaccharides content (water-soluble and -insoluble) and a bacterial culture method to determine the biofilm biomass (dry weight) and population. Furthermore, the amount of eDNA in the matrix was evaluated [47]. Briefly, after 67 h of formation, the biofilms were washed by immersion in wells containing sterile 0.89% NaCl solution. Each biofilm (disc) was transferred to a glass tube containing 1 mL of saline. Then, the walls of each tube were washed with 1 mL of saline solution. The glass tubes with biofilms/discs were placed in a Beaker, and the set was sonicated in a water bath for 10 min (model CD-4820, Kondentech Digital, São Carlos, Brazil). Afterward, the surfaces of each disc were scraped with the aid of a sterile metal spatula, taking care to remove any remaining biofilm from each disc. The volume of each biofilm suspension (2 mL) was transferred to a new 15 mL tube. Then, each glass tube was washed with 3 mL of saline solution, which was transferred to the tube containing the initial 2 mL, totaling 5 mL of biofilm suspension per biofilm/disc. Each biofilm suspension (5 mL) was sonicated through a probe at 7 w for 30 s. An aliquot of each suspension (100 µL) was used for serial dilutions (10^−1^ to 10^−5^) to determine the number of CFU/biofilm by plating on BHI agar plates (48 h, 37 °C, 5% CO_2_). Next, data CFU were transformed into log_10_ and analyzed compared to the vehicle control. One or two independent experiments were performed in duplicate. 

The remaining volume (4.9 mL) was centrifuged (3220× *g*, 20 min, 4 °C). The supernatant (with soluble extracellular matrix components) was transferred to a new tube (15 mL Falcon tube–supernatant), and the pellet (precipitate with the microbial cells and insoluble matrix components) was washed twice with 2.6 mL sterile Milli-Q water (3220× *g*, 20 min, 4 °C). The supernatants generated during the two washes were combined with the first supernatant obtained, totaling 10 mL, which was used to isolate and quantify water-soluble exopolysaccharides (6 mL plus 18 mL of 99% EtOH to precipitate the polysaccharides, followed by phenol-sulfuric colorimetric assay) [49], eDNA (500 µL) and proteins (500 µL) [9]. The pellet was suspended in 1 mL of Milli-Q water, of which 50 µL was used to quantify proteins and 950 μL to quantify insoluble dry weight (biomass), followed by the isolation and quantitation of water-insoluble exopolysaccharides (or alkali-soluble polysaccharides [48].

#### 4.7.2. Laser Scanning Confocal Fluorescence Microscopy 

For confocal microscopy analyses, biofilms were formed and treated with SPE-C18 (as described above), except that here, 1 μM Alexa Fluor™ 647-labeled dextran conjugate (absorbance/fluorescence emission maxima of 647/668 nm; Molecular Probes, Carlsbad, CA, USA) was added to the culture medium at the beginning of and during the development of the biofilms [50]. This method allows for the incorporation of labeled dextrans into exopolysaccharides during its synthesis process and matrix build-up. At 67 h of development, biofilms/discs were dip-washed into wells containing 0.89% NaCl and transferred to wells containing 0.89% NaCl solution and SYTO™ 9 (485/498 nm; Molecular Probes, Carlsbad, CA, USA), which is a green, fluorescent nucleic acid marker for detecting bacteria [50]. Each biofilm was scanned at three randomly chosen positions, and optical sectioning at each of these positions generated a series of confocal images. The image of the three-dimensional structure of these biofilms was performed using a Zeiss LSM 780 microscope (Zeiss, Jena, Germany) equipped with a Multialkali-PMT detector, 488 nm (SYTO9) and 561 nm (Alexa Fluor 647) laser, EC Plan-Neofluar objective of 20x, with a scale of 0.312 × 0.312 μm per pixel and increments of 1.5 μm. Images were acquired and analyzed using ZEN Blue 2.3 software for 3D reconstruction.

### 4.8. Growth Inhibition Curve for Selected Treatments Associated with Gene Expression Analysis

The fraction AcOEt_BRA/DF (250 µg/mL) showed activity against initial biofilm formation (24 h), and the molecule CsF (125 µg/mL) showed activity against pre-formed biofilm (48 h). Thus, they were tested in planktonic culture to determine *S. mutans* growth inhibition curve, using the cultures at the mid-log growth phase [51].

#### 4.8.1. Growth Inhibition Curve

In a 48-well polystyrene microplate, 150 μL of *S. mutans* inoculum (prepared as described above) was added to each well of the plate containing the treatments at the concentrations to be evaluated. Two wells were prepared to contain the inoculum and culture medium (TY + 1% glucose) and one containing only the culture medium (control for visual analysis of bacterial growth). An aliquot of the inoculum was seeded to determine the amount of CFU/mL at 0 h (before starting incubation and adding treatments-inoculum control). After incubation for 1 h, 2 h, 4 h, 6 h, and 24 h (37 °C, 5% CO_2_), visual observation and seeding of cultures on BHI agar plates were performed. For that, a 10 μL aliquot of the pure culture was seeded on a BHI agar plate, and 40 μL was removed from each well and transferred to microtubes containing 360 μL of 0.89% NaCl (dilution 1:10 *v*/*v*), followed by serial dilution and seeding on BHI agar plates. The plates were incubated (48 h, 37 °C, 5% CO_2_). After that, colony counts were performed for the treatment and vehicle control, and the calculation of the log number of CFU/mL of the treatment was compared to the vehicle control. Two experimental occasions were carried out, in duplicate, for the treatments and control tested (*n* = 2). At 2 h, 4 h and 24 h an aliquot of AcOEt_BRA/DF was diluted 1:1 (*v*/*v*) in 2.5% glutaraldehyde, and the same procedure was performed for CsF at 4 h and 24 h. The samples were stored in a refrigerator for scanning electron microscopy (SEM) analysis.

#### 4.8.2. Preparation of Cultures for SEM Analysis

Samples were diluted 1:11 *v*/*v* in 2.5% glutaraldehyde and were centrifuged (5 min, 15,294× *g*, 4 °C). Then, the supernatant was discarded, taking care not to detach the pellet, and 500 µL of 70% EtOH was added to the pellets, and the samples were incubated for 1 h at room temperature. Afterward, the samples were centrifuged (5 min, 15,294× *g*, 4 °C; Centrifugue 5430R, Eppendorf, Hamburg, Germany), the supernatant discarded, 500 µL of 90% EtOH was added to the pellets, and the samples were incubated for another 1 h at room temperature. Afterward, the samples were centrifuged (5 min, 15,294× *g*, 4 °C) and the supernatant was discarded. Then, the pellets were resuspended in 10 µL of absolute EtOH, and this volume was transferred to clean coverslips and placed in a 24-well plate. After complete evaporation of absolute EtOH, the plate was kept in a glass desiccator with silica until the analysis. Each sample was fixed with double-sided tape on the sample holder, and after, the samples were covered with carbon. The analysis using a high-resolution field emission electron microscope (MEV-FEG; JEOL, model JSM-7500F) with PC operating software PC-SEM v 2,1,0,3, equipped with secondary electron detectors, backscattered and chemical analysis (energy dispersive spectroscopy-EDS; Thermo Scientific, Ultra Dry model, USA) with NSS 2.3 operating software.

### 4.9. Inhibition of Growth by Different Compounds for Gene Expression Analysis 

Lastly, to explain the difference in survival profile between the compounds, *S. mutans* planktonic cells were treated with different agents with recognized targets to know which possible targets were involved in the observed biological activity. The following agents were used: compound 1771 [(5-phenyl-1,3,4-oxadiazol-2-yl)carbamoyl]methyl 2-{naphtho [2,1-b]furan-1-yl}acetate) (UkrOrgSynthesis, Ltd., Kiev, Ukraine, catalog n° PB25353228; purity not available), 4′ hydroxychalcone (C135) [(2E)-1-(4-hydroxyphenyl)-3-phenylprop-2-en-1-one) (AK Scientific, Inc., Union City, USA, catalog n° C135; 98% purity), myricetin (J10595) [3,5,7-trihydroxy-2-(3,4,5-trihydroxyphenyl)-4H-chromen-4-one] (AK Scientific, Inc., catalog n° J10595; 95% purity), *tt*-farnesol [(E,E)-3,7,11-trimethyl-2,6,10-dodecatrien-1-ol, trans,trans-3,7,11-trimethyl-2,6,10-dodecatrien-1-ol] (Sigma-Aldrich Co., St. Louis, MO, USA, catalog n° 46,193; 96% purity)], sodium fluoride (Sigma-Aldrich, catalog n° 71519), chlorhexidine digluconate solution (Sigma-Aldrich, catalog n^o^ C9394).

The agents and their concentrations were selected based on antimicrobial activity data previously tested in the laboratory [33,47,51]. The concentration of CHX was the same in mouthwashes commercialized for the control of biofilms, and sodium fluoride is the most seen in mouthwashes [52]. A starter culture was prepared as described above. For the inoculum, two 50 mL Falcon tubes were used to dilute the starter culture (1:20) using 2 mL of starter culture plus 38 mL TY + 1% glucose. When the appropriate OD was reached, the cultures were centrifuged (3220× *g*, 20 min, 4 °C), and the supernatants were discarded. Next, the pellet was resuspended with half the initial volume of TY + 1% glucose. This volume was divided into 12 tubes, then treatments were added, followed by incubation, but the time was different based on the survival curve (Table S4 in the Supplementary Materials). After incubation for the described time, an aliquot was removed for plating (to confirm the reduction in cell viability; Table S5 in the Supplementary Materials). The tubes were placed on ice for 15 min, centrifuged (3220× *g*, 20 min, 4 °C), the supernatants were discarded, and the pellets were resuspended with 1 mL of RNAlater (Ambion, Molecular Probes, Austin, TX, USA). Samples were frozen at −80 °C until RNA was isolated.

#### Gene Expression of *S. mutans*

The RT-qPCR (Reverse Transcription–quantitative Polymerase chain reaction) methodology included RNA isolation, cDNA synthesis, and gene expression analysis via qPCR of selected genes. Five specific genes were selected for expression profile analysis: genes associated with exopolysaccharides (*gtfB*, synthesis of insoluble glucans) and eDNA (*lrgA*) metabolism, acid stress tolerance (*atpD*), and acid and oxidative stress tolerance (*nox1*), with glycolysis (*eno*, enolase enzyme, fluoride target; Table 5) [6,51,53,54]. The 16S rRNA gene was included as an expression control (as a normalizer for the expression of specific genes) [55].

An optimized methodology for *S. mutans* was used to isolate the RNA, following a phenol-chloroform separation method, and the purification through the treatment with DNAse in column (Rneasy Micro Kit, Qiagen) and DNAse in solution (TURBO DNAse; Ambion) [56]. Afterward, spectrophotometry was used to evaluate the amount (ƞg/µL-OD260nm) and purity (OD 260/280 ratio) of the RNA samples (Nano-spectrophotometer DS-11+, Denovix). After purification, the RNA was evaluated for integrity through 1% agarose gel electrophoresis (Ultra Pure Invitrogen). There was no adequate RNA yield for the CHX-treated culture, and this sample was excluded from the analyses. Samples were kept at −80 °C until cDNA synthesis for RT-qPCR.

cDNA was synthesized (in triplicate per sample) using 0.25 µg of total RNA, and the High-Capacity cDNA Reverse Transcription kit (Thermo Fisher; catalog n° 4368814). Reactions containing all kit ingredients except the reverse transcriptase enzyme served as negative controls, determining whether there was DNA contamination. Reactions were incubated using the CFX96 Touch™ Real-Time PCR Detection System (Bio-Rad Laboratories, Hercules, CA, USA), using the protocol determined by the manufacturer, following the cycle: 10 min/25 °C, 120 min/37 °C, 5 min/85 °C, ∞/4 °C. Then, the samples were stored in a freezer at −20 °C until their dilution (1:5 for specific genes; 1:1000 for 16S rRNA gene and cDNA negative controls were not diluted) and used to quantify gene expression. The cDNA dilutions and negative controls were also stored in a −20 °C freezer.

The cDNA and negative controls were amplified by the CFX96 using specific primers from the literature and 2× SYBR PowerUp Green Master Mix (Thermo Fisher; catalog n° A25776), following previously determined protocols [54]. For each gene, a standard curve based on the purified PCR product of the target gene was included [57]. The reactions were incubated in the CFX96 thermocycler, using the following amplification cycle: 2 min/50 °C; 2 min/95 °C; 39 times: 0:15 min/95 °C; 0:30 min/58 °C and 1 min/72 °C; 0:15 min/95 °C; Melt Curve 60.0 °C to 95.0 °C: Increment 0.5 °C 0:05. It was observed that for the *gtfB* and *lrgA* genes, the amplification was inadequate using the SYBR Thermo (i.e., the efficiency was lower than 90%), and so, the reaction was carried out with the iQ^TM^ SYBR^®^ Green Supermix, Bio-rad (catalog n° 170-8882) and its cycle. This amplification was adequate, as per the MIQE246 guidelines [58]. The standard curves were used to transform the Quantification Cycle (Cq) values to relative numbers of cDNA molecules. Next, a fold difference was calculated per agent/treatment vs. the vehicle control for each gene.

### 4.10. Data Analyses

The data obtained were submitted to descriptive statistical analysis to compare groups with the vehicle. Data were organized in a database (Excel). The graphical representation of the results was performed using the statistical software Prism 8 (GraphPad Software). Data from antimicrobial activity, antibiofilm activity (long exposure time), and growth inhibition curve were analyzed by comparing the evaluated groups with vehicle control (independent variables) as CFU/mL count (dependent variable) and gene expression (fold-change). The biofilm characterization data included the response variables: biomass (mg), population (CFU/biofilm), and extracellular matrix components (ASP (µg), WSP (µg), and eDNA (ƞg)).

## Figures and Tables

**Table 1:** Samples of *C. sylvestris* from different Brazilian biomes.

Population ^a^	Sample Code	Variety ^b^	Biome	Plant Part	Geographic Location
Araraquara/SP	ARA/SP	L	Cerrado	Leaves	Associação Servidores Campus Araraquara–Ascar (21°49′08.7″ S 48°11′48.1″ W)
Araraquara/SP	ARA/SP	L	Cerrado	Fruits	Associação Servidores Campus Araraquara–Ascar (21°49′08.7″ S 48°11′48.1″ W)
Araraquara/SP	ARA/SP	S	Cerrado	Leaves	Associação Servidores Campus Araraquara–Ascar (21°49′08.7″ S 48°11′48.1″ W)
Araraquara/SP	ARA/SP	I	Cerrado	Leaves	Institute of Chemistry, UNESP (21°48′25.0″ S 48°11′33.2″ W)
Brasília/DF	BRA/DF	L	Cerrado	Leaves	University of Brasilia–UNB (15°45′49.4″ S 47°54′23.4″ W)
Presidente Venceslau/SP	PRE/SP	S	Atlantic Forest	Leaves	Km 622 of the Raposo Tavares Highway (21°51′36.0″ S 51°56′29.8″ W)
Presidente Venceslau/SP	PRE/SP	S	Atlantic Forest	Twigs	Km 622 of the Raposo Tavares Highway (21°51′36.0″ S 51°56′29.8″ W)

^a^ Four individuals sampled; ^b^ varieties based on botanical classification: L: var. *lingua*; S: var. *sylvestris*; I: intermediate morphology.

**Table 2:** Structure of flavonoids and clerodane diterpenes from *C. sylvestris*.

Molecules	Molecular Geometry	Reference
Flavonoid 4 (Quercetin-3-O-rutinoside–Rutin)	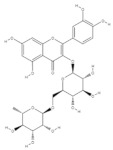	[28]
Caseargrewiin F	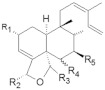	[17]
Casearin X		
Casearin J		

**Table 3:** *C. sylvetris* clerodane diterpenes and their substituents R1 to R5.

Casearins	R1	R2	R3	R4	R5
Caseargrewiin F	OBu	OAc	OAc	OH	H
Casearin X	OBu	OBu	OAc	OH	H
Casearin J	OMe	OBu	OAc	OH	OBu

**Table 4:** Formulations used to topically treat biofilms formed on sHA discs.

Treatments	Topical Treatment Time (min)
G3.3a—1000 µg/mL	1.5
G3.3a—1000 µg/mL + NaF 250	
G3.3a—500 µg/mL + F4.2b—500 µg/mL	
G3.3a—500 µg/mL + F4.2b—500 µg/mL + NaF 250	
NaF 250	
Vehicle	
AcOEt_BRA/DF—250 µg/mL	10
AcOEt_BRA/DF—250 µg/mL + NaF 250	
AcOEt_BRA/DF—1000 µ/mL	
AcOEt_BRA/DF—1000 µ/mL + NaF 250	
NaF 250	
CsF—125 µg/mL	10
CsF—125 µg/mL + NaF 250	
CsF—250 µg/mL	
CsF—250 µg/mL + NaF 250	
NaF 250	
Vehicle	

G3.3a denotes the fraction SPE-C18 Water/EtOH 40:60; F4.2b denotes the fraction SPE-C18 Water/EtOH 60:40; NaF 250 is 250 ppm sodium fluoride and diluent carrier 42.075% EtOH, 7.5% DMSO and 50% pH 6.0 buffer.

**Table 5:** Primers used for RT-qPCR.

Gene	GenBank Locus Tag	Sequence (forward and reverse)	Primer Concentration (ƞM)	Product Size (bp)	Reference
16 S rRNA		ACCAGAAAGGGACGGCTAAC	200	122	[6]
		TAGCCTTTTACTCCAGACTTTCCTG			
*gtfB*	SMU_1004	AAACAACCGAAGCTGATAC	250	90	
		CAATTTCTTTTACATTGGGAAG			
*nox1*	SMU.765	GGACAAGAATCTGGTGTTGA	250	91	[54]
		CAATATCAGTCTCTACCTTAGGC			
*atpD*	SMU.1528c	GGCGACAAGTCTCAAAGAATTG	250	115	
		AACCATCAGTTGACTCCATAGC			
*eno*	SMU_1247	GTTGAACTTCGCGATGGAGAT	250	150	[51]
		GTCAAGTGCGATCATTGCTTTAT			
*lrgA*	SMU_575c	GTCTATCTATGCTGCTATT	300	109	[53]
		AAGGACATACATGAGAAC			

**Figure A3:**
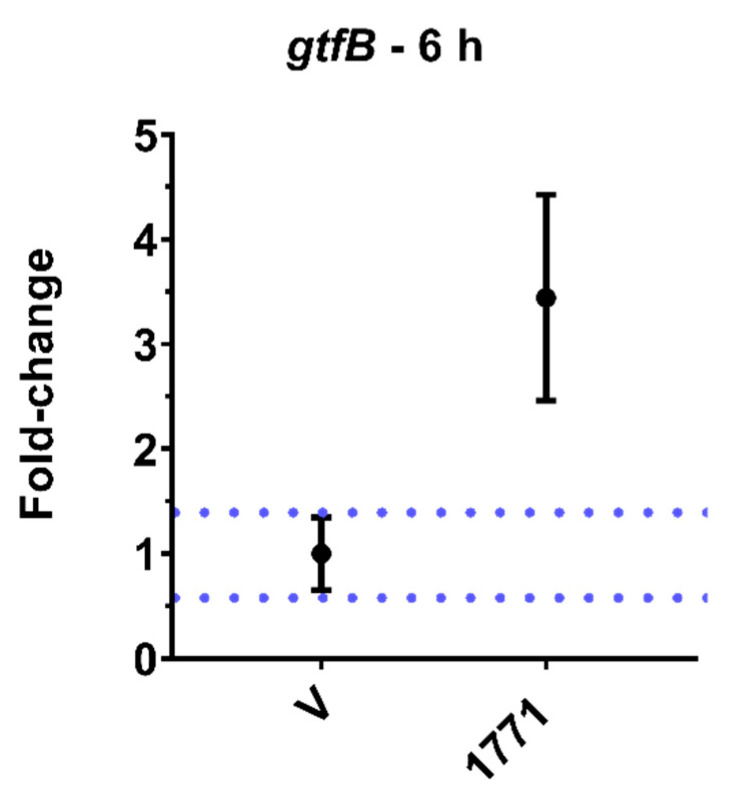
*S. mutans gtfB* expression of planktonic cultures. The central data are the mean, and the error bars are the confidence interval (95% CI). The dotted lines use vehicle data to compare the effectiveness of tested treatments.

## Data Availability

The data presented in this study are available on request from the corresponding author.

## Supplementary Materials

The following supporting information can be downloaded at: https://www.mdpi.com/article/10.3390/antibiotics12020329/s1, Table S1. Activity against initial biofilm formation (24 h) of fractions of *C. sylvestris* against *S. mutans*; Table S2. Activity against initial biofilm formation (24 h) of fractions of *C. sylvestris* against *S. mutans*; Figure S1. Samples of leaves and fruits of specimens collected from *C. sylvestris* showing anatomical details; Table S3. pH values of crude extracts of *C. sylvestris*; Table S4. The treatment agents used and their respective incubation were different based on the survival curve; Table S5. Cell viability reduction (log_10_ CFU/mL) of *S. mutans* cultures, Figure S2. Experimental design for topical treatment regimen in biofilms formed on sHA discs.
