# Supplementary material for: Effect of Extracts, Fractions, and Isolated Molecules of Casearia sylvestris to Control Streptococcus mutans Cariogenic Biofilm
Source: Antibiotics (Basel). 2023 Feb 4;12(2):329. doi: 10.3390/antibiotics12020329 (PMC9952592; doi:10.3390/antibiotics12020329)
Supplement: Supplementary file 1 [file antibiotics-12-00329-s001.zip › antibiotics-2128161-supplementary.pdf]

Effect of Extracts, Fractions, and Isolated Molecules of *Casearia sylvestris* to Control *Streptococcus mutans* Cariogenic Biofilm

Sabrina M. Ribeiro. Paula C. P. Bueno. Alberto José Cavalheiro. Marlise I. Klein

Supplementary Materials

Table S1. Activity against initial biofilm formation (24 h) of fractions of *C. sylvestris* against *S. mutans*

	ARA *lingua* leaves (Hex)	ARA *lingua-* leaves (AcOEt)	ARA *lingua* leaves (EtOH)	ARA *lingua*fruits (Hex)	ARA *sylvestris* leaves (Hex)	ARA *sylvestris* leaves (EtOH)	ARA intermediate leaves (EtOH)	BRA *lingua* leaves (Hex)	V	Sm
**Log$_{10}$ CFU/mL**	7.29	6.93	7.02	7.11	7.18	7.15	7.38	7.15	7.41	7.03
	7.26	7	6.99	7.15	7.15	7.1	7.27	7.19	7.49	7.1
	7.19	5.7	7.05	6.95	7.07	7.15	7.22	7.14	7.43	7.21
	7.21	5.6	7.11	7	7.02	7.19	7.21	7.07	7.41	7.14
	-	7.07	7.02	7.09	-	7.23	7.26	7.26	7.71	7.15
	-	7.11	7.05	7.13	-	7.24	7.22	7.25	7.79	7.14
**Mean**	7.24	6.57	7.04	7.07	7.1	7.17	7.26	7.18	7.21	7.15
**SD**	0.04	0.71	0.04	0.08	0.07	0.05	0.06	0.07	0.05	0.05
**%CV**	0.62	10.87	0.61	1.11	0.99	0.74	0.86	1.01	0.76	0.75
**CFU/mL**	1.94 x 10^{7}	8.50 x 10^{6}	1.05 x 10^{7}	1.30 x 10^{7}	1.50E x 10^{7}	1.42 x 10^{7}	2.39 x 10^{7}	1.41 x 10^{7}	2.60 x 10^{7}	1.08 x 10^{7}
	1.80 x 10^{7}	1.00 x 10^{7}	9.70 x 10^{6}	1.40 x 10^{7}	1.40 x 10^{7}	1.25 x 10^{7}	1.86 x 10^{7}	1.54 x 10^{7}	3.10 x 10^{7}	1.25 x 10^{7}
	1.54 x 10^{7}	5.00 x 10^{5}	1.12 x 10^{7}	9.00 x 10^{6}	1.18 x 10^{7}	1.40 x 10^{7}	1.65 x 10^{7}	1.37 x 10^{7}	2.70 x 10^{7}	1.62 x 10^{7}
	1.63 x 10^{7}	4.00 x 10^{5}	1.30 x 10^{7}	9.90 x 10^{6}	1.05 x 10^{7}	1.54 x 10^{7}	1.63 x 10^{7}	1.18 x 10^{7}	2.60 x 10^{7}	1.39 x 10^{7}
	-	1.18 x 10^{7}	1.05 x 10^{7}	1.22 x 10^{7}	-	1.69 x 10^{7}	1.80 x 10^{7}	1.82 x 10^{7}	5.10 x 10^{7}	1.42 x 10^{7}
	-	1.28 x 10^{7}	1.12 x 10^{7}	1.36 x 10^{7}	-	1.72 x 10^{7}	1.67E x 10^{7}	1.79 x 10^{7}	6.20 x 10^{7}	1.39 x 10^{7}
**Mean**	1.73 x 10^{7}	7.33 x 10^{6}	1.10 x 10^{7}	1.20 x 10^{7}	1.28 x 10^{7}	1.50 x 10^{7}	1.83 x 10^{7}	1.52 x 10^{7}	3.72 x 10^{7}	1.36 x 10^{7}
**SD**	1.78 x 10^{6}	5.53 x 10^{6}	1.12 x 10^{6}	2.05 x 10^{6}	2.05 x 10^{6}	1.82 x 10^{6}	2.88 x 10^{6}	2.50 x 10^{6}	1.55 x 10^{6}	1.81 x 10^{6}
**%CV**	10.31	75.45	10.16	17.15	15.96	12.08	15.68	16.49	41.66	13.31

The table shows mean and standard deviation (SD) log$_{10}$ CFU of biofilms treated by fractions obtained from lyophilized and dried extracts in a sample concentrator (methodology 1). Data from 1 experimental occasion. Growth control (no treatment) is represented as Sm for *S. mutans* and V for vehicle control (the concentration in each well being 5.26% EtOH and 0.94% DMSO).

Table S2. Activity against initial biofilm formation (24 h) of fractions of *C. sylvestris* against *S. mutans*

	**ARA *lingua* leaves (Hex)**	**ARA *lingua* leaves (AcOEt)**	**ARA *lingua* leaves (EtOH)**	**ARA *lingua* fruits (Hex)**	**ARA *sylvestris* leaves (Hex)**	**ARA *sylvestris* leaves (EtOH)**	**ARA intermediate leaves (Hex)**	**ARA intermediate leaves (EtOH)**	**BRA *lingua* leaves (Hex)**	**V**	**Sm**
**Log_{10} CFU/mL**	7.38	7.19	6.49	7.2	6.75	6.91	6.62	5.36	6.94	5.3	5.3
	7.3	7.21	6.52	7.23	6.79	6.94	6.57	5.2	6.9	5.6	5.48
	7.32	7.05	6.49	7.23	6.41	6.87	6.7	5.62	6.89	7.13	7
	7.49	7.08	6.56	7.11	6.52	6.92	6.64	5.64	6.96	7.03	7.11
	7.36	6.93	6.41	7.23	7.09	6.3	6.68	6.79	6.98	7.12	7.09
	7.38	6.89	6.32	7.08	7.27	6.34	6.69	6.81	6.92	7.09	7.08
**Mean**	7.37	7.06	6.47	7.18	6.8	6.71	6.65	5.9	6.93	6.54	6.51
**SD**	0.07	0.13	0.08	0.07	0.33	0.31	0.05	0.71	0.04	0.85	0.87
**%CV**	0.9	1.84	1.3	0.94	4.81	4.54	0.72	12.03	0.52	13.03	13.36
**CFU/mL**	2.40 x 10^{7}	1.54 X 10^{7}	3.10 X 10^{6}	1.60 X 10^{7}	5.60 X 10^{6}	8.20 X 10^{6}	4.20 X 10^{6}	2.30 X 10^{5}	8.80 X 10^{6}	2.00 X 10^{5}	2.00 X 10^{5}
	2.00 x 10^{7}	1.62 X 10^{7}	3.30 X 10^{6}	1.70 X 10^{7}	6.10 X 10^{6}	8.70 X 10^{6}	3.70 X 10^{6}	1.60 X 10^{5}	7.90 X 10^{6}	4.00 X 10^{5}	3.00 X 10^{5}
	2.10 X 10^{7}	1.11 X 10^{7}	3.10 X 10^{6}	1.70 X 10^{7}	2.60 X 10^{6}	7.40 X 10^{6}	5.00 X 10^{6}	4.20 X 10^{5}	7.70 X 10^{6}	1.35 X 10^{7}	1.00 X 10^{7}
	3.10 X 107	1.19 X 10^{7}	3.60 X 10^{6}	1.30 X 10^{7}	3.30 X 10^{6}	8.30 X 10^{6}	4.40 X 10^{6}	4.40 X 10^{5}	9.20 X 10^{6}	1.06 X 10^{7}	1.29 X 10^{7}
	2.30 X 10^{7}	8.50 X 10^{6}	2.60 X 10^{6}	1.70 X 10^{7}	1.23 X 10^{7}	2.00 X 10^{6}	4.80 X 10^{6}	6.10 X 10^{6}	9.50 X 10^{6}	1.31 X 10^{7}	1.22 X 10^{7}
	2.40 X 10^{7}	7.80 X 10^{6}	2.10 X 10^{6}	1.20 X 10^{7}	1.86 X 10^{7}	2.20 X 10^{6}	4.90 X 10^{6}	6.40 X 10^{6}	8.40 X 10^{6}	1.24E X 10^{7}	1.19 X 10^{7}
**Mean**	2.38 X 10^{7}	1.18 X 10^{7}	2.97 X 10^{6}	1.53 X 10^{7}	8.08 X 10^{6}	6.13 X 10^{6}	4.50 X 10^{6}	2.29 X 10^{6}	8.58 X 10^{6}	9.61 X 10^{6}	7.57 X 10^{6}
**SD**	3.87 X 10^{6}	3.46 X 10^{6}	5.35 X 10^{5}	2.25 X 10^{6}	6.19 X 10^{6}	3.15 X 10^{6}	4.76 X 10^{5}	3.07 X 10^{6}	7.14 X 10^{5}	5.81 X 10^{6}	5.66 X 10^{6}
**%CV**	16.23	29.24	18.05	14.68	76.55	51.41	10.59	133.94	8.32	60.52	74.81

The table shows mean and standard deviation (SD) log_{10} CFU of biofilms treated by fractions obtained from dry extracts in the sample concentrator and dried in a fume hood (methodology 2). Data from 1 experimental occasion. Growth control (no treatment) is represented as Sm for *S. mutans* and V for vehicle control (the concentration in each well being 5.26% EtOH and 0.94% DMSO).

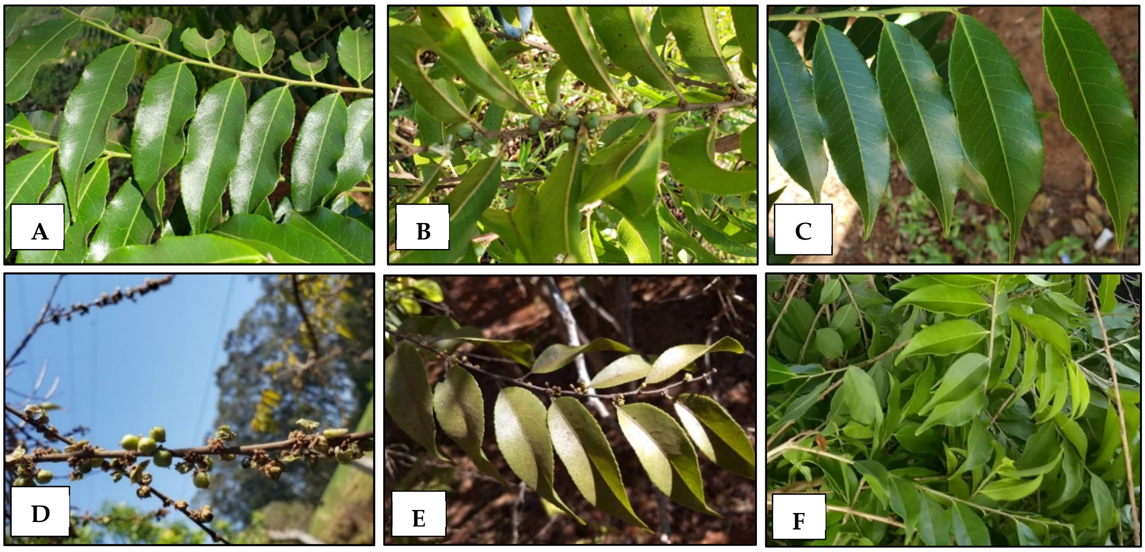

Figure S1. Samples of leaves and fruits of specimens collected from *C. sylvestris* showing anatomical details. Leaf and fruit specimens from specimens collected from *C. sylvestris*. **A.** Sample of ARA/SP var. *sylvestris*. **B.** Sample of ARA/SP var. *lingua*. **C.** Sample of ARA/SP var. intermediate. **D.** Sample of fruits from ARA/SP var. *lingua*. E. Sample of BRA/DF var. *lingua*. F. Sample of PRE/SP var. *sylvestris*.
Source: Author's personal archive.

Table S3. pH values of crude extracts of *C. sylvestris*

Sample Code	Variety	Plant part	Work Solution Concentration	pH value when diluted (1:1) in 2.5M phosphate buffer pH 6.2	pH value when diluted (1:1) in 2.5M phosphate buffer pH 6.8
**ARA/SP**	*lingua*	leaves	12 mg/mL	6.996	-
**ARA/SP**	*lingua*	fruits	12 mg/mL	7.064	-
**ARA/SP**	*sylvestris*	leaves	12 mg/mL	6.852	7.101
**ARA/SP**	intermediate	leaves	12 mg/mL	6. 527	7.03
**BRA/DF**	*lingua*	leaves	12 mg/mL	6.515	7.076
**PRE/SP**	*sylvestris*	leaves	18 mg/mL	6.882	-
**PRE/SP**	*sylvestris*	twigs	18 mg/mL	6.945	-
**Vehicle**		-		7.039	-

To check the pH of each extract and vehicle (no microorganism) the stock solutions were diluted 1:1 in PBS (2.5 mM phosphate buffer. pH 6.2) and then added to glass tubes containing TY + 1 % sucrose (concentration of extracts 500 μg/mL). The pH was measured using a digital pH meter. Crude extracts with 0.2 pH difference *vs.* vehicle (in blue) were adjusted by diluting in PBS (2.5 mM phosphate buffer) pH 6.8.

Table S4. Treatment agents used and their respective incubation were different based on the survival curve

	Volume (μL)								
	**CHX**	**C135**	**AcOEt_ BRA/DF**	***tt*-farnesol**	**CsF**	**1771**	**J10595**	**Fluoride (NaF)**	**V**
Compound concentration	0.12%	250 μg/mL	250 μg/mL	125 μg/mL	125 μg/mL	7.812 μg/mL	500 μg/mL	250ppm	0 (V)
Stock volume 2 mg/mL 1771. 1 mg/mL CsF. 2 mg/mL AcOEt and 15 mg/mL others in 84.15% EtOH + 15% DMSO. Sodium fluoride 5000 ppm. CHX 20%	9.00	25.05	187.49	12.53	187.49	5.85	50.03	75.00	0.00
Vehicle volume (V) - 42.075% EtOH + 7.5% DMSO (μL) + 50% phosphate buffer pH 6	187.49	137.39	0.00	162.42	0.00	175.79	87.43	187.49	187.49
Culture medium volume (μL)	1303.51	1337.56	1312.51	1325.06	1312.51	1318.36	1362.54	1237.51	1312.51
Total volume per tube	1500	1500	1500	1500	1500	1500	1500	1500	1500
incubation time	10 min	10 min	1 h	1 h	4 h	6 h	6 h	6 h	-[&]

EtOH: Ethanol; DMSO: dimethylsulfoxide; CHX: chlorhexidine; CsF: Caseargrewiin F. & There was the vehicle control for each incubation time with specific compounds.

Table S5. Cell viability reduction (log_{10} CFU/mL) of *S. mutans* cultures

	**Inoculum**	**CHX 0.12%**	**C135 250 µg/mL**	**V 10 min**	**AcOEt_BRA/ DF 250 µg/mL**	***tt*- farnesol 125 µg/mL**	**V 1 h**	**CsF 125 µg/mL**	**V 4 h**	**NaF 250 ppm**	**Compound 1771 7.812 µg/mL**	**Myracetin (J105951) 500 µg/mL**	**V 6 h**
**Log10**	9.45	5.83	6.18	9.11	7.56	6.87	9.08	7.45	8.29	8.91	8.22	6.94	8.33
**CFU/mL**	9.41	5.98	6.31	9.18	7.48	6.83	9.12	7.39	8.34	8.86	8.21	7.09	8.29
**Mean**	9.43	5.91	6.25	9.14	7.52	6.85	9.1	7.42	8.32	8.89	8.22	7.02	8.31
**SD**	0.03	0.1	0.09	0.05	0.06	0.03	0.03	0.04	0.03	0.03	0	0.1	0.03
**CFU/mL**	2.80×10^{9}	6.80×10^{5}	1.52×10^{6}	1.28×10^{9}	3.60×10^{7}	7.40×10^{6}	1.21×10^{9}	2.80×10^{7}	1.97×10^{8}	8.10×10^{8}	1.65×10^{8}	8.80×10^{6}	2.12×10^{8}
	2.56×10^{9}	9.50×10^{5}	2.05×10^{6}	1.50×10^{9}	3.00×10^{7}	6.80×10^{6}	1.32×10^{9}	2.46×10^{7}	2.18×10^{8}	7.30×10^{8}	1.64×10^{8}	1.22×10^{7}	1.93×10^{8}
**Mean**	2.68×10^{9}	8.15×10^{5}	1.79×10^{6}	1.39×10^{9}	3.30×10^{7}	7.10×10^{6}	1.27×10^{9}	2.63×10^{7}	2.08×10^{8}	7.70×10^{8}	1.65×10^{8}	1.05×10^{7}	2.03×10^{8}
**SD**	$1.70E \times 10^{8}$	1.91×10^{5}	3.75×10^{5}	1.56×10^{8}	4.24×10^{6}	4.24×10^{7}	7.78×10^{7}	2.40×10^{6}	1.48×10^{7}	5.66×10^{7}	7.07×10^{5}	2.40×10^{6}	1.34×10^{8}

S. mutans cultures were treated by different agents and the respective exposure times to treatments. The table shows mean and standard deviation of data from 1 experimental occasion. Vehicle control is represented as V for each incubation time. SD denotes standard deviation.

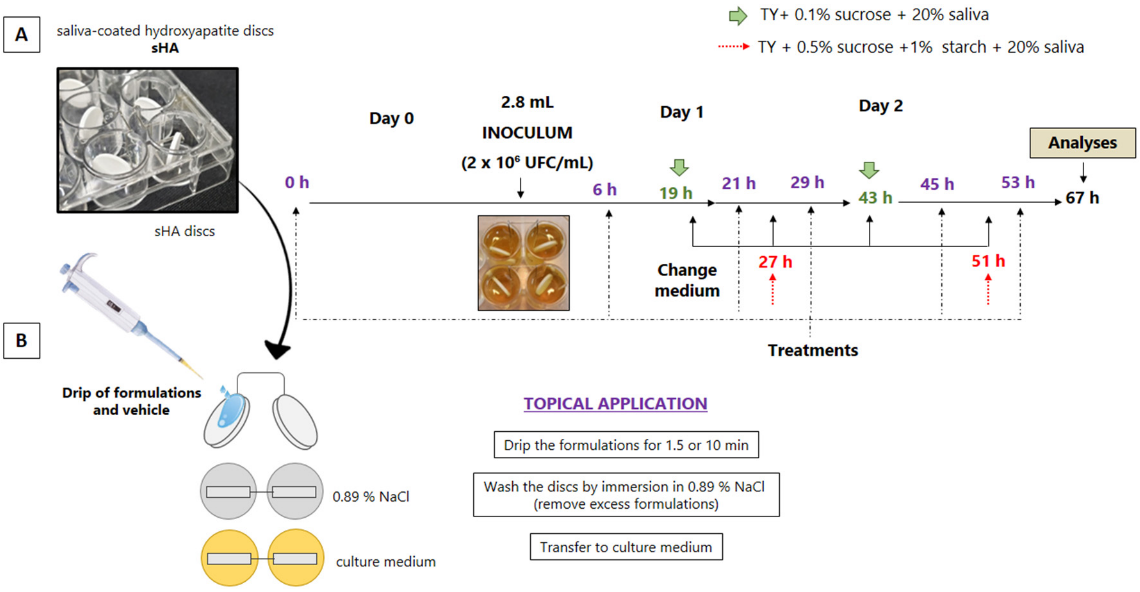

Figure S2. Experimental design for topical treatment regimen in biofilms formed on sHA discs. **A.** The sHA discs were treated at 0 (salivary pellicle before incubation with *S. mutans*) and after 6, 21, 29, 45, and 53 h of biofilm development. At 67 h of development, the biofilms were processed for analysis. The pH of the spent medium was evaluated at 19, 27, 43, 46, 51, and 67 h. **B.** Illustration of topical treatment of salivary film-coated hydroxyapatite discs. Topical treatment consisted of dripping the formulations (or vehicle control).
